# Analyzing biomarker discovery: Estimating the reproducibility of biomarker sets

**DOI:** 10.1371/journal.pone.0252697

**Published:** 2022-07-28

**Authors:** Amir Forouzandeh, Alex Rutar, Sunil V. Kalmady, Russell Greiner

**Affiliations:** 1 Department of Computing Science, University of Alberta, Edmonton, Canada; 2 Department of Pure Math, University of Waterloo, Waterloo, ON, Canada; 3 Canadian VIGOUR Centre, University of Alberta, Edmonton, Canada; 4 Alberta Machine Intelligence Institute, Edmonton, Canada; Taipei Medical University, TAIWAN

## Abstract

Many researchers try to understand a biological condition by identifying *biomarkers*. This is typically done using univariate hypothesis testing over a labeled dataset, declaring a feature to be a biomarker if there is a significant statistical difference between its values for the subjects with different outcomes. However, such sets of proposed biomarkers are often not reproducible – subsequent studies often fail to identify the same sets. Indeed, there is often only a very small overlap between the biomarkers proposed in pairs of related studies that explore the same phenotypes over the same distribution of subjects. This paper first defines the *Reproducibility Score* for a labeled dataset as a measure (taking values between 0 and 1) of the reproducibility of the results produced by a specified fixed biomarker discovery process for a given distribution of subjects. We then provide ways to reliably estimate this score by defining algorithms that produce an over-bound and an under-bound for this score for a given dataset and biomarker discovery process, for the case of univariate hypothesis testing on dichotomous groups. We confirm that these approximations are meaningful by providing empirical results on a large number of datasets and show that these predictions match known reproducibility results. To encourage others to apply this technique to analyze their biomarker sets, we have also created a publicly available website, https://biomarker.shinyapps.io/BiomarkerReprod/, that produces these *Reproducibility Score* approximations for any given dataset (with continuous or discrete features and binary class labels).

## 1 Introduction

Improved understanding of a disease can lead to better diagnosis and treatment. This often begins by finding “biomarkers”, which is a generic term referring to “a characteristic that is objectively measured and evaluated as an indicator of normal biological processes, pathogenic processes, or pharmacologic responses to a therapeutic intervention”[1]. Typically, these are individual features (*e.g.*, expression values of specific genes [2, 3]) that follow different distributions (*e.g.*, have different mean values) in diseased patients versus healthy controls.

**Fig 1:**
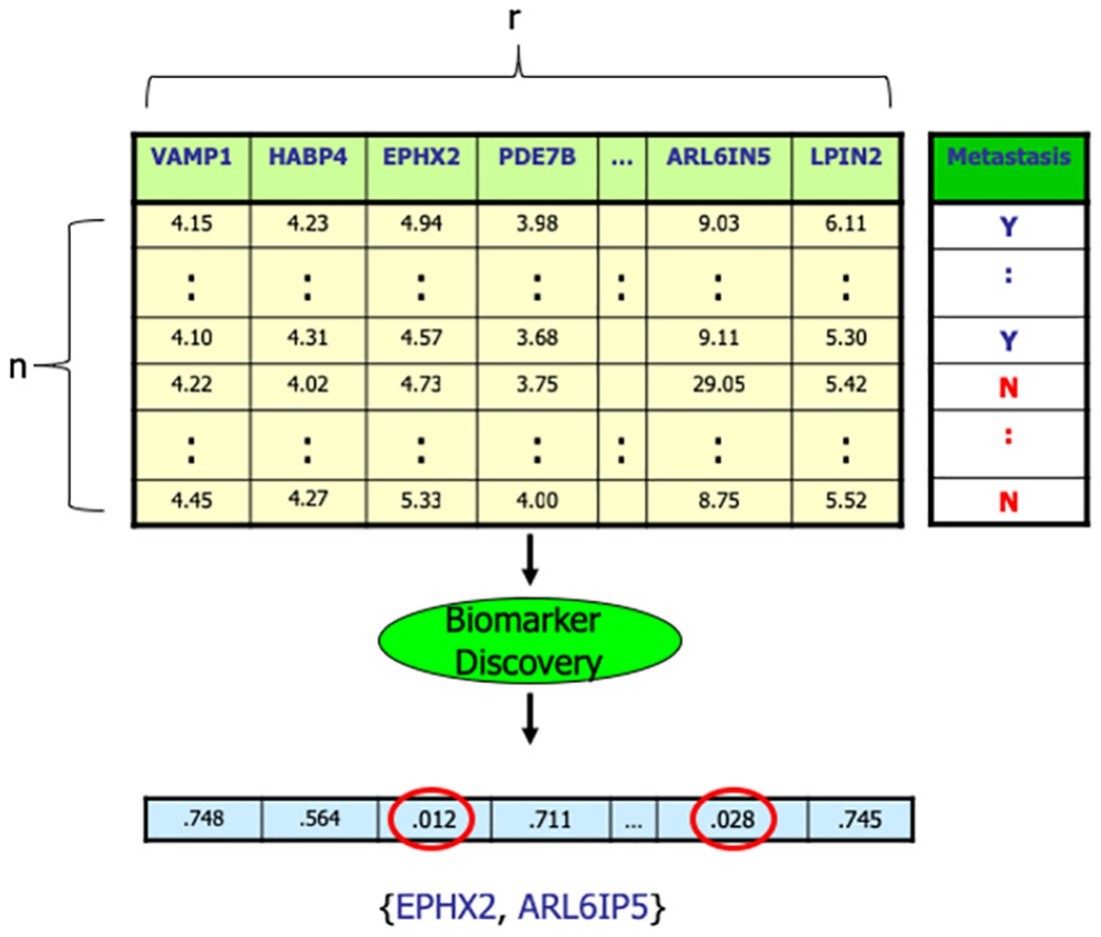
Data matrix, showing t-test p-values for each (shown) feature for the GSE 7390 dataset [14], with respect to the group outcome (here “Metastasis” for breast cancer). The circled features, with *p*<0.05, are (purported) biomarkers. For notation: We will refer to each of the first *r* columns of the matrix as a “feature”; these are often called “(independent) variables”. We refer to the final column as a “outcome”–*e.g.*, case versus control (shown here as Y versus N)–these are often called “labels”, “dependent variables”, “groups”, “phenotypes” or “classes”. Finally, we will use “subject” to refer to each row of that matrix; these are sometimes called “instances” or “samples”.

Sometimes, biomedical researchers can identify candidates for biomarkers based on their existing knowledge of the disease etiology and/or cellular pathways. This is done by seeking features that are causally related to the disease (*e.g.*, phenylketonuria is caused by mutations in the single gene PAH [4]) or a symptom of it (*e.g.*, Hemoglobin A1C for monitoring the degree of glucose metabolism in diabetes [5]). This paper, however, focuses on the use of statistical tools to discover and evaluate biomarkers, which is typically based on a dataset—a matrix whose rows each correspond to a subject (say a person) and each column corresponds to a feature (*e.g.*, clinical measure, or the expression value of a gene), and with a final column providing the outcome (*e.g.*, a binary outcome distinguishing case versus control); see Fig 1.

These “biomarker discovery studies” (also known as “association studies”) then attempt to determine which of the features (columns) differ significantly among distinct outcomes. Two standard examples of such studies are the “Genome Wide Association Studies” (GWASs), over a set of SNPs [6]; and the “Gene Signature Studies”, over gene expression values [7]. Typically, this involves first computing a representative statistic for each feature (*e.g.*, for continuous entries, running a t-test based on the mean and variance of the case versus control), and then declaring a feature to be a biomarker if the resulting MCC-corrected *p*-value is below 0.05 [8], where we use “MCC” (Multiple Comparison Corrections) as a generic term, which includes both False Discovery Rate (FDR) correction, and Family-Wise Error (FWE) Correction. (Section B.I in S1 Appendix discusses some of the subtleties here, especially with respect to features).

In some situations, the researchers then validate these biomarkers using a biological or medical process (*e.g.*, based on knock-out or amplification studies [9, 10]). Other studies validate the proposed biomarkers based on existing biological knowledge. A third class of projects instead use the potential biomarkers to create a computational model–perhaps to learn a classifier [11–13]–and then measure that down-stream model (perhaps based on its accuracy on a held-out set) and declare the biomarkers to be useful if that model scores well.

A great many papers, however, simply publish the list of purported biomarkers without providing validation for this set; see Section B.2 in S1 Appendix. This paper focuses specifically on this case. We address this limitation by providing a falsifiable (statistical) claim about such sets of biomarkers, which suggests a way to validate the proposed biomarker sets.

While some biomarkers are causally related to the associated outcome, this can be difficult to establish (often requiring instrumented studies [15]); but fortunately, in many situations, it may be sufficient for the features to be *correlated* with the phenotype. Here, an ideal biomarker discovery process would identify all-and-only the features that are *consistently* correlated with the associated disease, in that its presence (or absence or …) alone supports that disease. This argues that a proposed biomarker is good if it was *reproducible*—*i.e.*, that the biomarkers found in one study, would appear in many (ideally, all) future studies that explore this disease.

This has motivated the use of independent test sets to check the validity of the earlier findings. Unfortunately, many papers report this is not the case–*i.e.*, that relatively few biomarkers appear across multiple studies. For example, while the breast cancer studies by van’t Veer *et al.* [2] (resp., Wang *et al.* [3]) reported signatures with 70 (resp., 76) genes, these two sets had only 3 genes in common. Ein-Dor *et al.* [16] notes this in another situation: “Only 17 genes appeared in both the list of 456 genes of Sorlie *et al.* [17] and the 231 genes of van’t Veer *et al.* [2]; merely 2 genes were shared between the sets of Sorlie *et al.* and Ramaswamy *et al.* [18]. Such disparity is not limited to breast cancer but characterizes other human disease datasets (Alizadeh *et al.* [19]) such as schizophrenia (Miklos and Maleszka [20])”. Many others [21–23] report similar findings. Indeed, Begley and Ellis [24] report that only 6 of 53 published findings in cancer biology could be confirmed; which Wen *et al.* [25] notes is “a rate approaching an alarmingly low 10% of reproducibility”. Moreover, a 2016 Nature survey [26], of over 1500 scientists, found that 70% of researchers have tried but failed to reproduce another scientist’s experiments, and 52% thought there was a significant ‘crisis’ of reproducibility.

There are many possible reasons for this.

1. Each study should consider the *same well-defined “distribution” over instances*—*e.g.*, over the same distribution of ages and genders, etc. If the study attempts to distinguish case from control, then the two sub-populations should only differ in a single characteristic. Unfortunately, matching cases and controls over all possible features is often not achievable.
2. A second issue is with the precise notion of what “*reproducible*” means. Is it a property of a *specific biomarker*, or of *a set of biomarkers*? There is no clear choice for an optimal objective measure. This is especially problematic when dealing with multi-factorial diseases, where the outcomes correspond to a disjunction over many sub-diseases [27]. See also Section B.3 in S1 Appendix.
3. A final important issue is the impact of *sample size*. Many studies have a relatively low number of subjects, which increases the probability of finding both false negatives and false positives.

Our analysis assumes that the researchers have addressed (1) by running carefully designed, well-specified studies. Further, we also assume that there is no uncertainty in the outcomes with respect to its clinical or biological definition. We will provide a precise measure of reproducibility (2), as well as some specific implementations, and show empirically how this measurement varies with sample size (3).

In this paper, we assume that biomarkers are stand-alone features. Note each feature could be a pre-defined combination of single features (*e.g.*, the average expression values of the genes associated with a pre-defined signalling pathway—see gene enrichment [28]) or networks of genes associated with high loadings of principal component and univariate Pearson correlation values (see PC-corr [29]); but we are not considering *learning* combinations. By a *Biomarker Discovery* process BD(⋅), we mean a function that takes as input a labeled data matrix D of *n* subjects over a set of *r* features, each labeled with its outcome, and identifies a subset of proposed biomarkers; see Fig 1. We will formally define a *Reproducibility Score*, RS(*D*, BD), to quantify the “reproducibility” of the set of proposed biomarkers produced by the biomarker discovery process: *viz.*,

$$\begin{array}{l} \text{the}\;\text{average}\;\text{Jaccard}\;\text{score}\;\text{between}\;\text{these}\;\text{proposed}\;\text{biomarkers}\;\text{BD}(D),\\ \text{and}\;\text{those}\;\text{produced}\;\text{by}\;\text{running}\;\text{the}\;\text{same}\;\text{BD}\;\text{process}\\ \text{over}\;\text{another}\;\text{comparable}\;\text{dataset}\;\text{drawn}\;\text{from}\;\text{the}\;\text{same}\;\text{distribution} \end{array}$$ (1)

where two datasets are comparable if they have the same number of subjects from each outcome. (Section B.3 in S1 Appendix discusses some subtle issues related to “reproducibility”.) In order to estimate the reproducibility score in practice, we construct two approximations: an overbound and an underbound. We then provide empirical tests over many datasets, with a focus on *t*-tests as the main biomarker discovery process. We provide many examples for both microarray data (with continuous values) and SNP data (with discrete values) to provide practical evidence for the effectiveness of these approximations. Researchers can use this framework to estimate the reproducibility of the potential results of their biomarker discover study. A low reproducibility score suggests that these biomarkers may not be accurate, potentially because the dataset used is too small, the dataset is too heterogenous, or the biomarker discovery algorithm is not suitable for the dataset. To help users evaluate the quality of their proposed biomarker sets, we have also produced a publicly available website, https://biomarker.shinyapps.io/BiomarkerReprod/, that, given a labeled dataset, computes these estimates of the Reproducibility Score, with respect to any of a variety of biomarker discovery algorithms.

**Outline:** Section 2 formally defines the Reproducibility Score (RS) and describes the challenges of estimating this measure. We then define two approximations for RS: an overbound and an underbound. It also describes some of the standard biomarker discovery algorithms. Section 3 describes extensive empirical studies over many datasets—microarray and mRNAseq data (with continuous values) and SNP data (with discrete values), focusing on a standard Biomarker Discovery process BD(⋅), to confirm the effectiveness of these approximations. Section 4 summarizes some future work and the contributions of this paper. In the Supplementary Information, Section B in S1 Appendix discusses various notes: a glossary of the various technical terms used, how Biomarker Discovery differs from standard (supervised) Machine Learning, different notions of “reproducibility”, how a combination of a pair of features might be important for a predictive task, even if neither, by itself, is important (towards explaining why it can be so difficult to find biomarkers); etc. Section C in S1 Appendix presents results from other empirical studies, which explore how the RS varies with the type of MCC correction used (including “none”), the p-value threshold, the size of the dataset and the number of iterations of the approximation algorithms. Finally, the approximations we present are motivated by two heuristics (Heuristics 7 and 9). Section D in S1 Appendix presents arguments that motivate these heuristics, and also provide additional empirical evidence that support them.

We close this section by motivating the need for an objective measure for evaluating the quality of a set of biomarkers (Subsection 1.1), then overviewing some earlier studies that discuss the issue of reproducibility in biomarker discovery and/or provide approaches that could be beneficial when dealing with such problems (Subsection 1.2).

### 1.1 Motivation for evaluating biomarker sets

To motivate the need for evaluating association studies, consider first *predictive studies*, which use a labeled dataset, like the one shown at the top of Fig 1, to produce a predictive model (perhaps a decision tree, or a linear classifier) that can be used to classify future subjects–here, into one of the two classes: Y or N. In addition to the learned classifier, the researchers will also compute *a meaningful estimate of its quality*–*i.e.*, of the accuracy (or AUROC, Kappa Score, etc.) of this classifier on an independent hold-out set [30], or the results of *k*-fold cross-validation over the training sample.

By contrast, many association studies report only a set of purported biomarkers, but provide no falsifiable claim about the accuracy of these biomarkers. Many meta-reviews claim that a set of biomarkers is problematic if they are not reproduced in subsequent studies [16, 31–33]. Given that biomarkers should be reproducible, we propose evaluating a biomarker set based on its reproducibility score. An accurate estimate of this score can help in at least the following three ways:

1. Researchers can compare various different “comparable” biomarker discovery algorithms to see which produces the biomarker set that is most reproducible. Here, “comparable” corresponds to the standard practice of only considering discovery tools that impose the same criterion, such as the same *p*-value, or only considering features that exhibit the same minimum fold-change. This type of analysis may help to determine errors within the biomarker discovery process.
  Moreover, we will see that MCC-correction, while useful in removing false-positives, can be detrimental to the goal of producing reproducible biomarkers; similarly, there is no reason to insist on *p* < 0.05 for the statistic test used.
2. A low reproducibility score suggests that few of the proposed biomarkers will be found in another dataset, and highlights the potential that these proposed biomarkers may not be accurate. This could motivate researchers to consider a dataset that is larger, to focus on a more homogenous population, or perhaps consider another biomarker discovery technique.
3. Finally, there are many meta-reviews [21, 34, 35] that note the lack of repeatability in many biomarker discovery papers, and question whether the techniques used are to blame. One way to address this concern is to require that each published paper include both the purported set of biomarkers, and also an estimate of its reproducibility score. The same way a prediction study’s “5-fold cross validation” accuracy tells the reader how accurate the classification model should be on new data, this biomarker-discovery reproducibility score will inform the reader whether to expect another study, on a similar dataset, will find many of the same biomarkers. Note that we should view the reproducibility score as necessary for considering a proposed model, but not sufficient–*i.e.*, it might rule-out a proposed discovery model, but should not be enough to rule-in a model.

For these reasons, we provide an easy-to-use, publicly available webapp https://biomarker.shinyapps.io/BiomarkerReprod/ that anyone can use to produce meaningful estimates of the reproducibility of a set of biomarkers. (The underlying code is also available, from https://github.com/amirfrz/BMDA).

### 1.2 Related work

There have been many pairs of studies that have each produced biomarkers for the same disease or condition, but found little or no overlap between the two lists of purported biomarkers. Many papers have discussed this issue–some describing this problem in general [16, 33, 35], and others exploring specific examples [8, 32]. These papers suggest different reasons for the problem, such as the heterogeneous biological variations in some datasets [16, 33] or problems in the methods used that may lead to non-reproducible results [36, 37].

In particular, Zhang *et al.* [33] challenge the claim that the non-reproducibility problem in microarray studies is due to poor quality of microarray technology, by showing that inconsistencies occur even between technical replicates of the same dataset. They also show that heterogeneity in cancer pathology would further reduce reproducibility.

Ein-Dor *et al.* [16] also show the inconsistencies between the results of subsamples of a single dataset, demonstrating that the set of (gene) biomarkers discovered is not unique. They explain that there are many genes correlated with the group outcomes, but the empirical correlations change for different (sub)samples of instances. These two papers motivate our need for tools that can effectively estimate the reproducibility–such as the ones presented here.

Several projects [35–37] have attempted to formally analyse this problem. Ein-Dor *et al.* [35] describe a method, Probably Approximately Correct (PAC) sorting, that estimates the minimum number of instances needed for a desired level of reproducibility. As an example, this worst-case analysis proves that, to guarantee a 50% overlap between different gene lists for breast cancer, each dataset needs to include at least several thousand patients. This suggests poor repeatability results when using small sample sizes, which is consistent with our results for datasets with smaller sample sizes; see Subsection 3, especially Fig 4.

The goal of the MicroArray Quality Control (MAQC) project [36] was to address the problems and uncertainties about the microarray technology that were caused by the observation that different studies (of the same phenotype) often found very different biomarkers.

They suggest that the common approach of using just t-test *p*-values (especially with stringent *p*-values) can lead to poor reproducibility, which motivated them to consider methods like fold-change ranking with a non-stringent *p* cutoff, which they demonstrate leads to more reproducible gene sets. In a follow-up, Guo *et al.* [37] found similar results by using the same procedures for another dataset. However, Klebanov *et al.* [38] later show that these MAQC project results do not prove that using t-tests is necessarily unsuitable–*i.e.*, just because another method (here fold-change) can generate more reproducible results, does not mean that it is performing better; as an extreme, the algorithm that declares every gene is a biomarker (think *p* = 1.0), is completely reproducible. They demonstrate these points by using a set of simulation studies (where they know the “true biomarkers”), and use either t-test or fold-change to propose potential biomarkers. These studies found that the t-test approach performed much better than the fold-change, in terms of recall (sensitivity). These results motivated us to use the t-test approach (rather than fold-change) as our main BD algorithm–which we use for all of our empirical experiments.

Our approximation algorithms use a type of re-sampling to bound reproducibility. Below we summarize several other studies that similarly deal with re-sampling and biomarker discovery. Some studies provide ways to better estimate the true statistical significance, but do not provide a framework for evaluating empirical reproducibility—*e.g.*, Gagno *et al.* [39] used bootstrap resampling to estimate 95% confidence intervals and *p*-values for an internal assessment of their findings (related to breast cancer survival), Chitpin *et al.* [40] proposed a resampling-based method to better estimate the false discovery rate in chromatin immunoprecipitation experiments, and Pavelka *et al.* [41] proposed a resampling-based hypothesis testing algorithm that provides a control of the false positive rate for identification of differentially expressed genes. Furthermore, Alshawaqfeh *et al.* [42] and Zhao and Li [43] suggested methods for consistent biomarker detection in high-throughput datasets where evaluation was based on common biomarkers among the two resampled sets–*i.e.*, they are considering the false positives but not false negatives. (By contrast, our use of Jaccard score involves both.) Other studies had different goals–*e.g.*, Ma *et al.* [44] used resampling in a permutation test, to evaluate the predictive power of the identified gene set based on the accuracy of down-stream classification task; recall however that our goal is to evaluate the reproducibility of the biomarkers directly (not downstream). Filosi *et al.* [45] propose methods for evaluating the stability of reconstructed biological networks in terms of inference variability due to data subsampling; we however are focusing on the reproducibility of the individual biomarkers. Hua *et al.* [46] conducted a simulation study to compare the *ranking performance* of several gene set enrichment methods; by contrast, our approach considers the SET of biomarkers, not the ranking, and is over several real-world datasets (not just simulated ones). Note that none of these used re-sampling techniques to bound the expected replicability of the set of biomarkers found by some discovery algorithm, nor to demonstrate the validity of those bounds.

## 2 Materials and methods

### 2.1 Formal description

As illustrated in Fig 1, a “Biomarker Discovery” algorithm, BD(⋅), takes as input a dataset *D* of *n* subjects, each described by *r* features *F* = {*f*_1_, …, *f*_*r*_} and labeled with a binary outcome, and returns a subset *F*′ ⊂ *F* of purported biomarkers.

Typically, each *f* ∈ *F*′ differs in some significant way between each class. To be more precise, let $x_{i}^{j}$ be the value of the *i*^*th*^ feature of the *j*^*th*^ subject, and ℓ^(*j*)^ be the outcome of the *j*^th^ subject (which is either + or -). Then a class difference means that the set $\left\{x_{i}^{j}\;|\;\ell^{\left(j\right)}=+\right\}$ of values of the *i*^th^ feature of the diseased individuals is significantly different from the values of that feature over the healthy individuals, $\left\{x_{i}^{j}\;|\;\ell^{\left(j\right)}=-\right\}$. For simplicity, we will assume that these ${\left\{x_{i}^{j}\right\}}_{i,j}$ values are either all continuous (such as height, or the expression value of a gene), or all discrete (such as gender, or the genotype of a SNP). Subsection 2.2 below will describe several such biomarker discovery algorithms.

As noted in Eq 1, the *Reproducibility Score* RS(*D*, BD) quantifies the “reproducibility” of the set of proposed biomarkers BD(*D*) corresponding to running the biomarker discovery algorithm over the labeled dataset *D*. Here, we assume that the values of each feature $x_{.}^{j}$, for each outcome *c*, are generated independently from a fixed distribution (*i.e.*, “i.i.d.”)

$$\begin{array}{c} p_{i,c}\left(v\right)\;=\;P_{}\left(\;x_{i}^{j}=v\;|\;\ell^{\left(j\right)}=c\;\right). \end{array}$$

Here and in general, we use *P* (⋅) to refer to either a probability density for continuous variables, or a probability mass for discrete variables. Note these are just the marginal distributions: we do not assume that the various features are independent from one another.–*i.e.*, this does not necessarily correspond to Naive Bayes [30]. We will view $\vec{p}\left(\cdot \right)={\left[p_{i,c}\left(\cdot \right)\right]}_{i,c}$ as the matrix of these *r* × 2 different distributions, and let ${\vec{p}}^{\left[n_{+},n_{-}\right]}\left(\cdot \right)$ be the distribution for sampling *n* = *n*_+_ + *n*_−_ instances independently from this distribution, where $n_{+}\in Z^{+}$ instances are drawn from the distribution [*p*_1,+_(⋅), …, *p*_*r*,+_(⋅)] associated with positive outcomes, and similarly $n_{-}\in Z^{+}$ instances from the distribution [*p*_1,−_(⋅), …, *p*_*r*,−_(⋅)] associated with negative outcomes. Then for datasets $D^{\prime },D^{\prime \prime }∼{\vec{p}}^{\left[n_{+},n_{-}\right]}\left(\cdot \right)$ sampled independently, we define

$$\begin{array}{c} {\text{RS}}^{*}\left(\;\vec{p}\left(\cdot \right),\;\left[n_{+},n_{-}\right],\;\text{BD}(\cdot )\right)\;=\;E\left[\;J\left({\text{BD}}_{}\left(D^{\prime }\right),\;{\text{BD}}_{}\left(D^{\prime \prime }\right)\;\right)\;\right] \end{array}$$ (2)

where the Jaccard score of two sets

$$\begin{array}{c} J\left(A,\;B\;\right)\;=\;\frac{\left|\;A\;\cap \;B\;\right|}{\left|\;A\;\cup \;B\;\right|} \end{array}$$ (3)

is the ratio of the intersection to the union of these sets—hence *J*(*A*, *B*) ranges from 0 to 1, and is 1 if and only if *A* = *B* ≠ {}, and is 0 if and only if these sets are disjoint. (We define this to be 0 if *A* = *B* = {}.) Note that the Jaccard score is only one possible measure to evaluate the degree of overlap of gene signatures; Section C.4 in S1 Appendix discusses a slightly different measure that is sometimes used to measure the reproducibility of a set of biomarkers. See also Shi *et al.* [47] for a comprehensive overview of these measures.

Of course, we do not know $\vec{p}\left(\cdot \right)$, and so we use an empirical distribution $\hat{p_{D}}\left(\cdot \right)$, determined based on context from the dataset *D*, to produce the approximation

$$\begin{array}{c} \text{RS}\left(\;D,\;\text{BD}\right)\;=\;{\text{RS}}^{*}(\;\hat{p_{D}}\left(\cdot \right),\;\left\langle D\right\rangle ,\;\text{BD}(\cdot )) \end{array}$$ (4)

that estimates the reproducibility of the biomarker set BD(*D*), where the notation 〈*D*〉 = [|*D*^+^|, |*D*^−^|] refers to the pair of sizes of the positive and negative subjects in *D*—corresponding to [*n*_+_, *n*_−_]. Note that this reproducibility score deals with the *sets* of biomarkers that are produced by the BD(⋅) function, and not any specific biomarker.

Of course, Eq 4 suggests the obvious bootstrap sampling algorithm [48]. Empirically, however, we found that it did not perform well (see Section C.1 in S1 Appendix)–motivating the algorithms described in Subsection 2.3.

### 2.2 Biomarker discovery algorithms: BD(⋅)

We now discuss various approximation algorithms for biomarker discovery. As our goal is to illustrate the reproducibility issues with respect to the standard approach, we focus on that standard approach: where the biomarker discovery is based on independent two-sample *t*-tests, perhaps with some multiple comparison correction. This use of t-tests implicitly assumes that the feature values, for each outcome, are normally distributed. However, it is easy to adapt these algorithms to use other statistical tests, that do not make this distributional assumption.

See also Limitations in Section 4.

Recall that we are considering two types of datasets, depending on whether its feature values (the $x_{i}^{j}$ mentioned above) are continuous or discrete. However, for datasets with categorical values—SNPs in our analysis–we use a simple preprocessing step, which precedes all the BD(⋅) algorithms described here, to convert each categorical value to a real number: here converting each SNP feature, which ranges over the values { AA, Ab, bb }, to the real-values { 0, 1, 2 }, corresponding to the number of minor alleles (“b”) in the genotype. This allows us to view each such dataset as one with continuous values.

We assume that the real values of each feature, for each outcome, follows a normal distribution, which might be different for the different outcomes, and so we use an independent two-sample *t*-test for all of our empirical experiments. Recall that the test statistic is given by

$$\begin{array}{c} \text{t}\;=\;\frac{{\bar{X}}_{+}\;-\;{\bar{X}}_{-}}{{\bar{s}}_{p}\cdot \sqrt{\frac{1}{n_{+}}\;+\;\frac{1}{n_{-}}}}\;{\bar{s}}_{p}\;=\;\sqrt{\frac{(n_{+}-1)\;{\bar{s}}_{+}^{2}\;+\;(n_{-}-1)\;{\bar{s}}_{-}^{2}}{n_{+}\;+\;n_{-}-2}}. \end{array}$$ (5)

where *n*_+_ and *n*_−_ are the number of instances with positive and negative outcomes, respectively, and with empirical means ${\bar{X}}_{-}$ and ${\bar{X}}_{+}$ and empirical variances ${\bar{s}}_{+}^{2}$ and ${\bar{s}}_{-}^{2}$.

Note the biomarker discovery process essentially performs a single statistical test for each feature. As the number of features is often large–often tens-of-thousands, or more–many projects sought ways to reduce the chance of false discoveries; a standard way to do this is through some MCC method. We therefore consider biomarker discovery algorithms described as BD_*t*,*p*, *χ*_(*D*), where the *t* in the subscript refers to the 2-sided *t*-test, the *p* for the *p*-value used, and *χ* to the MCC method. Our canonical example is BD_*t*, 0.05, *BH*_(*D*), with *p* = 0.05, and *χ* = BH to the Benjamini/Hochberg correction [49]. This notation makes it easy to consider many variants –- *e.g.*, adjusting the *p*-value used for the statistical test, whether it is applying another multiple testing correction, or none, etc. See Section C.2 in S1 Appendix for more details.

### 2.3 Algorithms that approximate the reproducibility score

As we have the dataset *D* with *n* ≈ [*n*_+_, *n*_−_] labeled instances, we can directly compute BD(*D*). To compute RS(*D*, BD(⋅)), however, we also need to produce one (or more) similar datasets *D*′, each with [*n*_+_, *n*_−_] subjects drawn from the same (implicit) distribution $\vec{p}\left(\cdot \right)$ that generated *D* (with the same number of positive and negative instances), but which is presumably disjoint from *D*. While we do not have such *D*′’s, and so cannot directly compute the Reproducibility Score, we show below how to compute an overbound and an underbound of RS(*D*, BD(⋅)).

#### 2.3.1 Overbound: oRS

**Fig 2:**
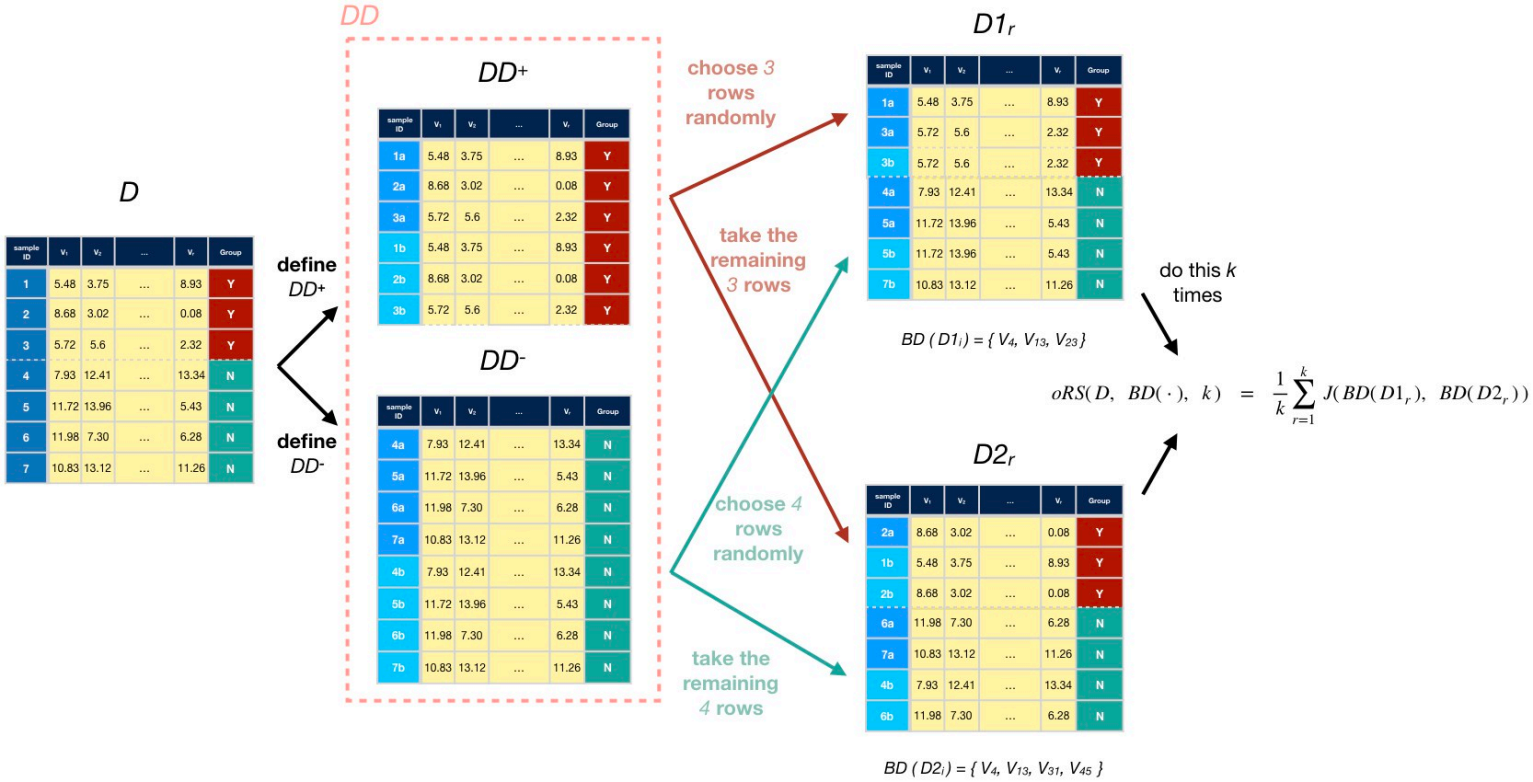
Computing oRS. Diagram showing oRS’s process of generating pairs of subsets for a dataset *D* (*k* times), then using those to compute oRS(D, BD(⋅), k).

The oRS(*D*, BD(⋅), *k*) procedure produces (an estimate of) an overbound for RS(*D*, BD(⋅)), by making it *easier* for a feature to be selected to be in both purported biomarker sets. In this algorithm, *D* is our given fixed dataset, BD(⋅) is the given biomarker discovery algorithm, and *k* is a parameter to determine the number of trials when computing the overbound. (Here, and below, see the Glossary in Section A in S1 Appendix for a summary of the algorithms and their arguments.) The oRS algorithm first defines a size-2*n* dataset *DD* that contains two copies of each subject in *D*, of course with the same outcome both times. It then randomly partitions this *DD* into two disjoint size-*n* datasets *D*_1_ and *D*_2_, balanced by outcome. Here, each partition is with respect to the *list* of elements, so it will include duplicates. To insure that the resulting datasets are balanced, oRS first split *DD* into *DD*^+^ and *DD*^−^, where *DD*^+^ are the cases and *DD*^−^ the controls. It then forms $D_{1}^{+}$ by randomly drawing 1/2 of *DD*^+^, and $D_{1}^{-}$ by randomly drawing 1/2 of *DD*^−^, then merges $D_{1}=D_{1}^{+}\cup D_{1}^{-}$; see Fig 2. The dataset *D*_2_ is then formed from the remaining subjects of *DD* not included in *D*_1_. oRS then runs BD(⋅) on the datasets *D*_1_ (resp., *D*_2_) to produce two sets of biomarkers, and computes the Jaccard score for this pair of biomarker sets: *J*(BD(*D*_1_), BD(*D*_2_)). It then repeats this double-split-BD-Jaccard process *k* times, then returns the average of these *k* values:

$$\begin{array}{c} \text{oRS}\left(\;D,\;\text{BD}\left(\cdot \right),\;k\right)\;=\;\frac{1}{k}\sum_{i=1}^{k}J\left(\text{BD}(D1_{r}),\;\text{BD}(D2_{r})\;\right) \end{array}$$ (6)

where each dataset pair [*D*1_*r*_, *D*2_*r*_] is created independently using the above procedure.

For each *r*, as we expect *D*1_*r*_ to overlap with *D*2_*r*_, it is relatively likely that any *D*1_*r*_-biomarker will also be a *D*2_*r*_-biomarker (more likely than if *D*1_*r*_ was disjoint from *D*2_*r*_), which means we expect the associated Jaccard score to be higher. This follows from the heuristic that, as two datasets have more common elements, we expect the number of biomarkers common to two datasets, to increase–*i.e.*,

$$\begin{array}{llll} \text{if} & A_{1},A_{2},B_{1},B_{2}\sim {\vec{p}}^{\left[n_{+},n\_\right]}(\cdot ) & \text{and} & \\ & \left|A_{1}\cap A_{2}\right| & \text{is}\;\text{larger}\;\text{than} & |B_{1}\cap B_{2}|,\\ \text{then}\;\text{we}\;\text{expect} & J(\text{BD}(A_{1}),\text{BD}(A2)) & \text{will}\;\text{be}\;\text{larger}\;\text{than} & J(\text{BD}(B_{1}),\text{BD}(B2)) \end{array}$$ (7)

*ceteris paribus*. Here, we view each of {*A*_1_, *A*_2_, *B*_1_, *B*_2_} as a set of *n* = *n*_+_ + *n*_−_*r*-dimensional instances. Note this relationship is simply a heuristic to motivate the algorithm–one that we expect to hold in practice. Section D in S1 Appendix provides some basic arguments, and empirical evidence, to support this claim.

We close with three quick observations:

1. **Expected Overlap:** We expect 50% of the instances to be duplicated in any given pair of datasets. See Lemma 2 in Section D.4 in S1 Appendix.
2. **Relation to Bootstrap Samples:** Here, each subject occurs exactly twice in each pair of datasets *D*1_*r*_ and *D*2_,*r*_. If we instead used bootstrap sampling (called bRS below), we expect many subjects would occur more often in the pair of datasets. (See Lemma 3 in Section D.4 in S1 Appendix.) Given Heuristic 7, this means we expect bRS’s Jaccard score here to be higher than for oRS’s; as oRS is already an overbound for the true score (RS), this means bRS would be a worse bound, as bRS ≥ oRS ≥ RS. This is why we use our “doubling approach” oRS rather than bootstrap sampling bRS, as oRS produces values that are smaller, but still remains an overbound, as desired. See Figs 7 and 8 and Section C.1 in S1 Appendix.
3. Relation to RS*: Our oRS approach is clearly related to RS* (Eq 2), as both compute the average Jaccard score of pairs of size-*n* datasets sampled from a distribution. They differ as (a) RS* uses the true distribution $\vec{p}\left(\cdot \right)$, while oRS uses only the estimate ${\hat{p}}_{D}\left(\cdot \right)$, (b) RS* is the *true* average while oRS is just the *empirical* average over *k* trials, and (c) RS* will draw *independent* datasets, but the oRS datasets will overlap.

#### 2.3.2 Underbound: uRS

The uRS(*D*, BD, *k*) procedure produces (an estimate of) an underbound for RS(*D*, BD(⋅)) by making it *harder* for a feature to be selected to be in both purported biomarker sets. First, observe that as [*n*_+_, *n*_−_] increases (keeping the *n*_+_-to-*n*_−_ ratio fixed, as we consider changing the size of the dataset), we expect the statistical estimates to be more accurate, and in particular, statistical tests for differences between the two classes will be correct more often. Hence, a statistical test will better identify the “true” biomarkers F* from a size-*n* subset *D*^(*n*)^, versus from a size-*n*/2 subset *D*^(*n*/2)^. Now consider two size-*n* datasets $D_{1}^{\left(n\right)}$ and $D_{2}^{\left(n\right)}$, and also two size-*n*/2 datasets $E_{1}^{\left(n/2\right)}$ and $E_{2}^{\left(n/2\right)}$. As $\text{BD}(D_{1}^{\left(n\right)})$ and $\text{BD}(D_{2}^{\left(n\right)})$ are each closer to F* than $\text{BD}(E_{1}^{\left(n/2\right)})$ and $\text{BD}(E_{2}^{\left(n/2\right)})$, we expect $\text{BD}(D_{1}^{\left(n\right)})$ and $\text{BD}(D_{2}^{\left(n\right)})$ to be closer to each other, than $\text{BD}(E_{1}^{\left(n/2\right)})$ and $\text{BD}(E_{2}^{\left(n/2\right)})$, which means we expect that

$$\begin{array}{c} J\left(\text{BD}(D1^{\left(n\right)}),\;\text{BD}(D2^{\left(n\right)})\;\right)\;\geq \;J\left({\text{BD}}_{}\left(E1^{\left(n/2\right)}\right),\;{\text{BD}}_{}\left(E2^{\left(n/2\right)}\right)\;\right). \end{array}$$

In general, given that

$$\begin{array}{c} {\hat{E}}^{\left(k\right)}\left[\;J\left({\text{BD}}_{}\text{(D1}{}^{\left(s\right)}\text{)},\;{\text{BD}}_{}\text{(D2}{}^{\left(s\right)}\text{)}\;\right)\;\right]\;\approx \;{\text{RS}}^{*}\left(\;\vec{p}\left(\;\cdot \right),\;s,\;\;\text{BD}(\cdot )\right) \end{array}$$ (8)

(where ${\hat{E}}^{\left(k\right)}\left[\cdot \right]$ is the empirical average over *k* samples), this argues that the RS* score should increase with the size *s* of the dataset–which suggests that

$$\begin{array}{llll} \text{if} & A_{1},A_{2},\sim {\vec{p}}^{sA}(\cdot ),B_{1},B_{2},\sim {\vec{p}}^{sB}(\cdot ) & \text{and} & \\ & sA=|A_{1}|=|A_{2}| & \text{is}\;\text{larger}\;\text{than} & sB=|B_{1}|=|B_{2}|,\\ \text{then}\;\text{we}\;\text{expect} & J(\text{BD}(A_{1}),\text{BD}(A2)) & \text{will}\;\text{be}\;\text{larger}\;\text{than} & J(\text{BD(}B_{1}),\text{BD}(B2)) \end{array}$$

Fig 4(a) presents empirical evidence, over 5 datasets, supporting this claim —showing that the Jaccard score increases as we increase the size *s* of the datasets. Section D in S1 Appendix provides some arguments, and additional empirical evidence (over hundreds of simulations), that further support this heuristic.

This motivates our underbound algorithm uRS(*D*, BD, *k*), which first partitions *D* into two disjoint size-*n*/2 subsets, *E*_1_ and *E*_2_, with balanced outcomes. It then computes *J*(BD(*E*_1_), BD(*E*_2_)) which, assuming Heuristic 9, is an underbound in expectation for RS(*D*, BD(⋅)). uRS does this partitioning *k* times, producing *k* different dataset pairs {[*E*_1,*r*_, *E*_2,*r*_]}_*r* = 1, …, *k*_, and returning the average Jaccard score, i.e.

$$\begin{array}{c} \text{uRS}\left(D,\;\text{BD}\left(\cdot \right),\;k\;\right)\;=\;\frac{1}{k}\sum_{r=1}^{k}J\left(\text{BD}(E_{1,r}),\;\text{BD}(E_{2,r})\;\right). \end{array}$$ (10)

See Fig 3.

**Fig 3:**
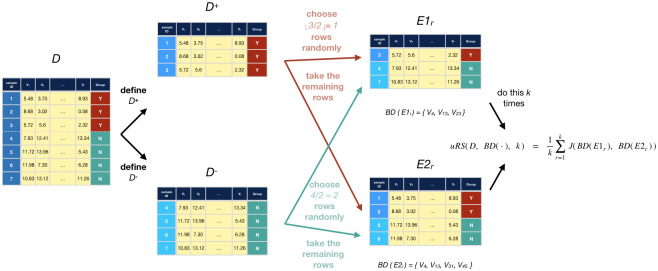
Computing uRS. Diagram showing uRS’s process of generating pairs of subsets for a dataset *D* (*k* times), then using those to compute uRS(D, BD(⋅), k).

### 2.4 Empirical study over various datasets

There are now many publicly-available datasets that have been used in association studies. Here, we use them to …

U1: Better understand what Jaccard scores are typical, for the standard BD(⋅) algorithms;
U2: Determine whether our predictions match the results of earlier meta-analyses; and
U3: Determine if our approximations are meaningful—*i.e.*, if (for reasonable values of *k*):
  $$\begin{array}{ccc} \text{uRS}(\;D,\;\text{BD},\;k) & \leq & \text{RS}(\;D,\;\text{BD}) \end{array}$$ (11)
  $$\begin{array}{ccc} \text{oRS}(\;D,\;\text{BD},\;k) & \geq & \text{RS}(\;D,\;\text{BD}) \end{array}$$ (12)

The next section will explicitly discuss all three issues. Of course, given only a single dataset *D* of size-*n*, we cannot compute, nor even estimate, the true value of RS(*D*, BD(⋅)). However, we can estimate RS(*D*^(*n*/2)^, BD(⋅)), where *D*^(*n*/2)^ is a size-*n*/2 (outcome-balanced) subset of *D*. In fact, uRS(*D*, BD(⋅), k) is a meaningful estimate of RS(*D*^(*n*/2)^, BD(⋅)); below we will use

$$\begin{array}{c} \hat{\text{RS}}\left(\;D^{\left(n/2\right)},\;\text{BD}\left(\cdot \right),\;k\right)\;=\;\text{uRS}\left(\;D,\;\text{BD}\left(\cdot \right),\;k\right)\;. \end{array}$$ (13)

We will then compare this $\hat{\text{RS}}\left(\;D^{\left(n/2\right)},\;\text{BD}(\cdot ),\;k\right)$ against uRS(*D*^(*n*/2)^, BD, *k*) and oRS(*D*^(*n*/2)^, BD, *k*) and to see whether the relations of Eqs 11 and 12 both hold, with respect to various size-*n*/2 subsets *D*^(*n*/2)^.

**Fig 4:**
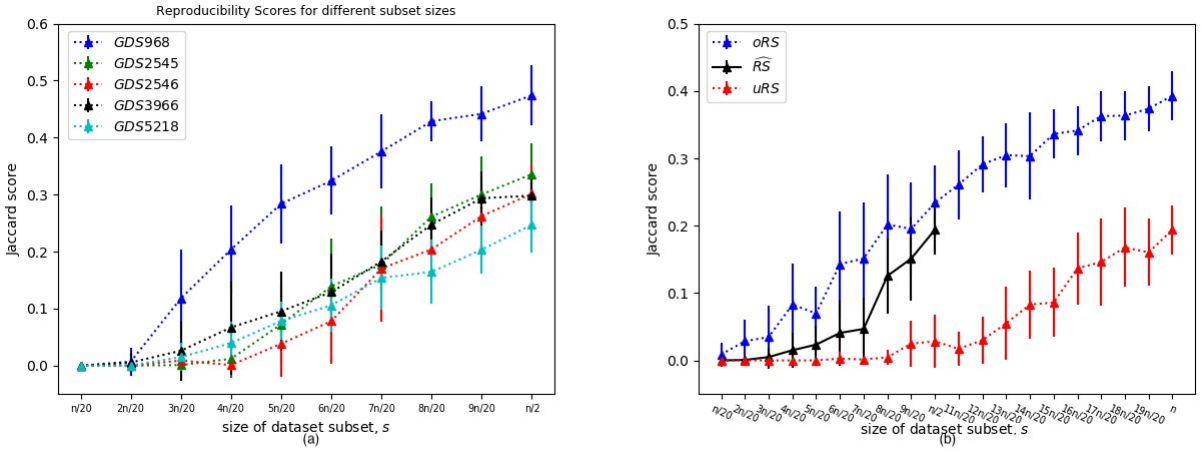
Reproducibility Scores for different subset sizes. (a) For each of the 5 datasets *D*, each point shows the average (± sd) Jaccard ${\hat{E}}^{\left(k\right)}\left[\;J\left({\text{BD}}_{}\left(D1^{\left(s\right)}\right),\;{\text{BD}}_{}\left(D2^{\left(s\right)}\right)\;\right)\;\right]$ over *k* = 20 pairs [*D*1^(*s*)^, *D*2^(*s*)^] of disjoint size-*s* subsets of *D*. Here, *n* is the size of the original dataset–note this can only go to *n*/2–and we are using the standard BD_*t*, 0.05, *BH*_. (b) Showing how the approximations relate to one another, and scale with the size *s* of the dataset. Here we are using subsets of the Metabric dataset, with *n* = 1654. We observed the same behavior for all datasets.

More generally, we can do this for any size-*s* subset *D*^(*s*)^ of *D* where *s* ≤ *n*/2. Here, we need a set of pairs of disjoint outcome-balanced subsets *D*′, *D*′′ ⊂ *D* where |*D*′| = |*D*′′| = *s* and *D*′∩*D*′′ = {}. For a fixed dataset *D*, and specified number $k\in Z^{>0}$, we can then plot these $\hat{\text{RS}}\left(\;D^{\left(s\right)},\;\text{BD}\left(\cdot \right),\;k\right)$ values along with oRS(*D*^(*s*)^, BD, *k*) and uRS(*D*^(*s*)^, BD, *k*), as a function of *s* to see their behaviour; see Fig 4(b), for the Metabric dataset. Our website https://biomarker.shinyapps.io/BiomarkerReprod/ also provides this visualization.

**Fig 5:**
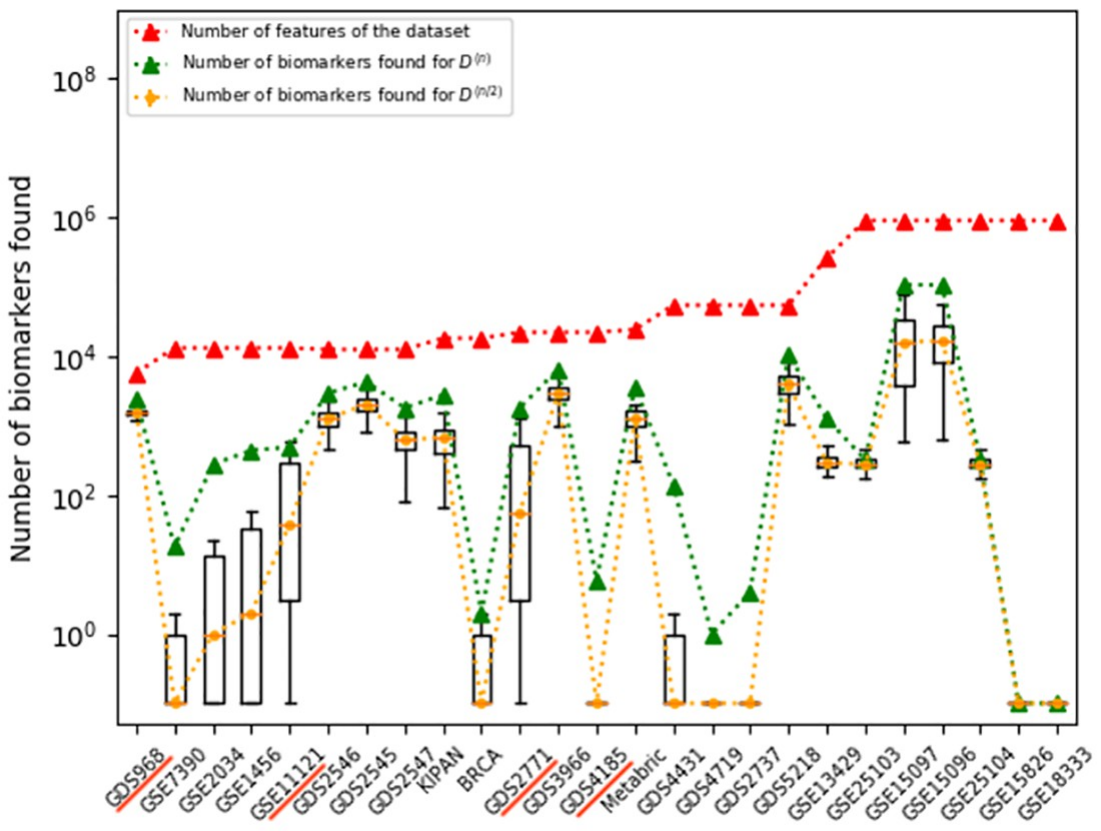
Number of biomarkers found for *D*^(*n*/2)^ compared to *D*^(*n*)^. Box+whiskers plots showing number of biomarkers found for *D*^(*n*/2)^ when using BD_*t*, 0.05, *BH*_ over *k* = 20 iterations for various dataset, compared to the number of biomarkers for *D*^(*n*)^, and to the number of features in each dataset. Note the y-axis is a log-scale. (Note we first changed all “0” values to “10^−1^”.) For details, see Tables 1 and 2.

We explored our approximations over **25 different real-world datasets, including 16 microarray datasets and 2 RNAseq datasets with continuous data, and 7 SNP datasets with categorical data** (see Table 1). This first set includes 4 of the gene expression datasets discussed in the Zou *et al.* [8] meta-analysis—each describing metastatic versus non-metastatic breast primary cancer subjects—to see if our method is consistent with their empirical results. We also included 11 other relatively-small gene expression datasets (with 19 to 187 subjects), focusing on human studies that had a binary class outcome from the GEO repository. To explore how our tools scale with size, we also included 3 other relatively large datasets, with 532 to 1654 subjects. As these were survival datasets, we set the binary outcome based on the median survival time (removing any subject that was censored before that median time). In addition to these 4+11+3 = 18 gene expression datasets (with real-valued entries), we also include 7 SNP datasets (with 39 to 164 subjects), with discrete values, also selected from human studies with binary class outcome s. Fig 5 plots the number of features and biomarkers found, using the BD_*t*, 0.05, *BH*_ algorithm, for each dataset—both *D*^(*n*)^ and *D*^(*n*/2)^.

**Table 1:** Results for all 25 datasets when using all the subjects. This table is sorted by sample size (#subjects)–corresponding to Fig 5. The first 18 entries are Gene Expression datasets (including the 4 “*”ed entries, from the Zou *et al.* [8] meta-study), and the final 7 are SNP datasets. Reproducibility Scores are shown in the form of mean ± standard deviation. The “(Majority %)” values are the percentage of the subjects in the dataset with the more common outcome–*e.g.*, 53% of the subjects in the GDS968 dataset are labeled “+” (for “long survival time”), and 82% of GSE7390 are labeled “−” (for “Non-Metastatic”).

Name	#subjects (Majority %)	#features	#biomarkers	uRS %	oRS %
GDS968 [50]	171 (53%)	5748	2506	47.5 ± 3.95	63.25 ± 0.76
GSE7390* [14, 51]	198 (82%)	13245	18	0 ± 0	5.15 ± 2.81
GSE2034* [3]	286 (67%)	13245	277	0 ± 0	12.6 ± 4.19
GSE1456* [52]	159 (78%)	13245	443	0 ± 0	13.6 ± 6.4
GSE11121* [53]	200 (86%)	13245	492	0.09 ± 0.2	13.4 ± 5.89
GDS2546 [54, 55]	167 (54%)	12553	2965	30.8 ± 4.82	49.0 ± 0.55
GDS2545 [54, 55]	171 (53%)	12558	4291	34.0 ± 4.09	54.58 ± 0.31
GDS2547 [54, 55]	164 (54%)	12579	1810	23.7 ± 5.86	42.66 ± 0.70
KIPAN [56]	532 (81%)	18271	2782	12.3 ± 4.91	34.2 ± 4.74
BRCA [56]	552 (95%)	18320	2	0 ± 0	2.5 ± 2.1
GDS2771 [57, 58]	187 (52%)	22215	1807	0.32 ± 0.64	31.68 ± 0.47
GDS3966 [59]	83 (63%)	22274	6554	31.7 ± 4.71	53.66 ± 0.27
GDS4185 [60, 61]	67 (58%)	22283	6	0 ± 0	11.29 ± 0.83
Metabric [62]	1654 (57%)	24368	3675	18.5 ± 3.86	39.8 ± 3.56
GDS4431 [63]	146 (53%)	54613	140	0 ± 0	18.85 ± 0.34
GDS4719 [64]	19 (53%)	54675	1	0 ± 0	7.02 ± 0.71
GDS2737 [65]	37 (57%)	54675	4	0 ± 0	10.58 ± 0.5
GDS5218 [66]	110 (56%)	54675	10700	24.0 ± 5.09	46.06 ± 0.29
GSE13429 [67]	39 (79%)	262314	1267	1.66 ± 0.44	21.4 ± 3.76
GSE25103 [68]	122 (92%)	908512	325	0.27 ± 0.14	3.94 ± 2.22
GSE15097 [69]	68 (59%)	909456	108224	4.9 ± 2.9	34.2 ± 5.64
GSE15096 [69]	69 (58%)	909457	106482	5.37 ± 2.9	33.4 ± 5.28
GSE25104 [68, 70]	122 (92%)	909547	326	0.27 ± 0.14	3.94 ± 2.22
GSE15826 [71]	164 (54%)	909549	0	0 ± 0	2.5 ± 0.58
GSE18333 [72]	82 (54%)	909606	0	0 ± 0	0 ± 0

## Results

We ran our suite of methods over the aforementioned 25 datasets, including 16 microarray datasets and 2 mRNAseq datasets, whose feature-values $\left\{x_{i}^{j}\right\}$ (recall each $x_{i}^{j}$ is the expression value of the *i*-th gene for the *j*-th subject; we log_2_-transformed the values from the mRNAseq datasets) and 7 were SNP datasets with categorical entries, *i.e.*, $x_{i}^{j}\in \left\{0,1,2\right\}$ is the number of minor alleles in the genotype for the *i*^th^ SNP for the *j*^th^ subject; see Table 1. Here, we use the standard BD_*t*, 0.05, *BH*_(⋅) biomarker discovery algorithm; see Section 2.2.

**Fig 6:**
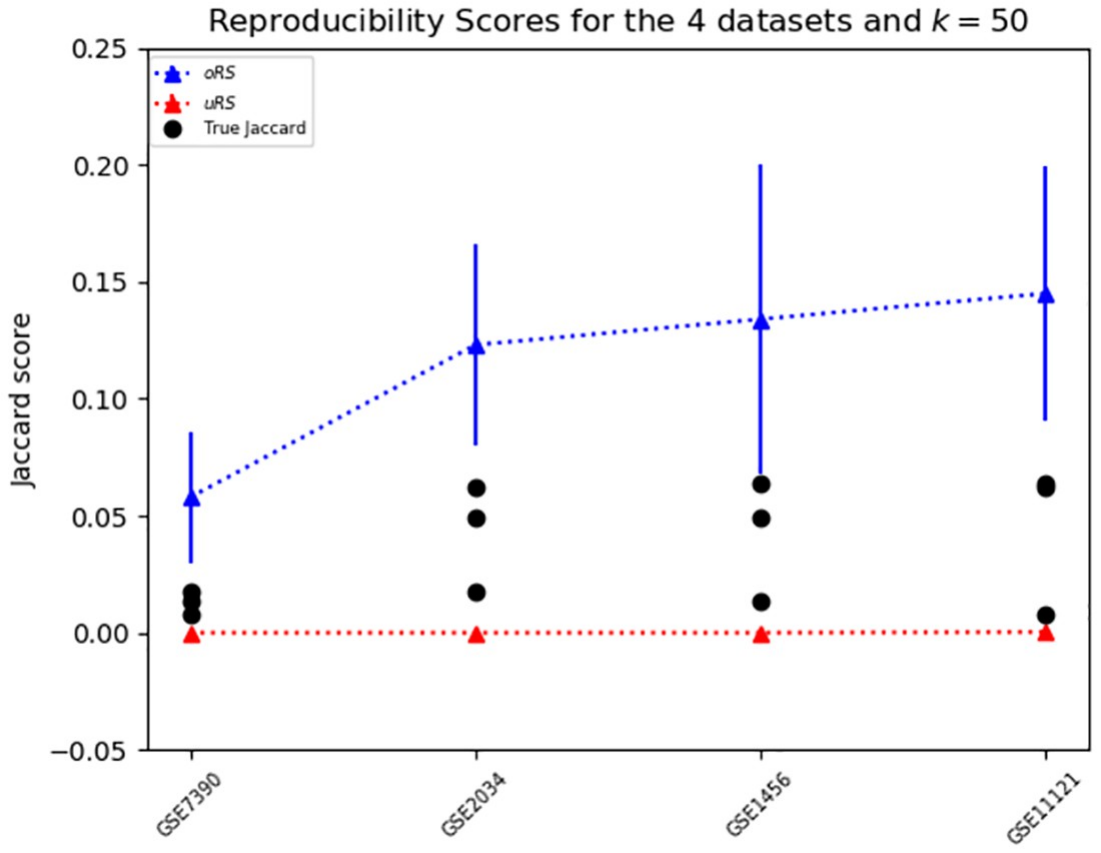
oRS and uRS compared to real Jaccard scores. Under-bound and over-bound for the 4 datasets from Zou *et al*. [8], as well as the true Jaccard score for each pair—3 numbers for each dataset, shown by black circles.

First, to address (U1) and (U2) in Section 2.4, we analyzed the 4 datasets mentioned in the Zou *et al.* [8] meta-analysis (see the 4 “*” rows of Table 1) and computed the {uRS(*D*, BD_*t*, 0.05, *BH*_, 50), oRS(*D*, BD_*t*, 0.05, *BH*_, 50)} values for each dataset *D*, as well as the actual Jaccard score for biomarkers for each pair of datasets. (We were not able to replicate the results reported for the 5^th^ dataset from that study, and so we excluded that one dataset from our analysis.) The results, in Fig 6, show that the Jaccard score for each pair is well within the bounds computed by our approximations, for each of the datasets in that pair—that is, the results for 4 × 3 = 12 ordered-pairs of datasets are consistent with our predictions. Note that we verified that our BD_*t*, 0.05, *BH*_ algorithm matched the original study by verifying that the PO scores (Eq 15 in Section C.4 in S1 Appendix) matched the ones that were originally published.

**Fig 7:**
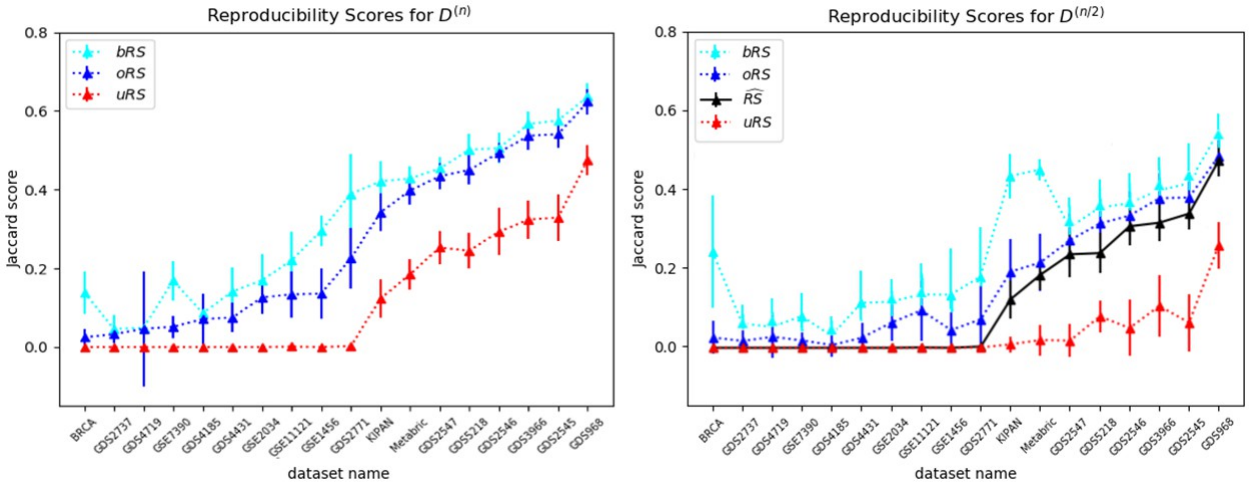
Empirical results for continuous datasets. Reproducibility scores (mean and standard deviation) for all 18 continuous datasets, both for complete datasets with *n* subjects (left) and for half-sized with $\frac{n}{2}$ subjects (right), for *k* = 50 iterations. The x-axes (for both plots) are sorted by the value of the over-bound for the *D*^(*n*)^ datasets. We see, in both, that the over-bound oRS is consistently higher than the under-bound uRS. Moreover, the right plot shows that the “truth” $\hat{\text{RS}}$ is also between uRS and oRS. (The bRS lines in the plots are based on the Bootstrap Overbound method; see Section C.1 in S1 Appendix).

To address (U3), we also analyzed the other 14 continuous datasets *D*, and computed the oRS and uRS values using *k* = 50 repetitions; see Fig 7[left]. We see that the overbound oRS is consistently larger than the underbound uRS—*i.e.*, uRS ≤ oRS—as claimed by Eqs 11 and 12. Fig 7[right] plots the corresponding values for the *D*^(*n*/2)^ datasets, that use only 1/2 of the dataset, using the same BD(⋅) algorithm and *k* = 50. It also plots the “true” $\hat{\text{RS}}\left(\;D^{\left(n/2\right)},\;\text{BD}\left(\cdot \right),\;k\right)$ values for the datasets. Again, we see that $\text{oRS}\geq \hat{\text{RS}}\geq \text{uRS}$.

**Fig 8:**
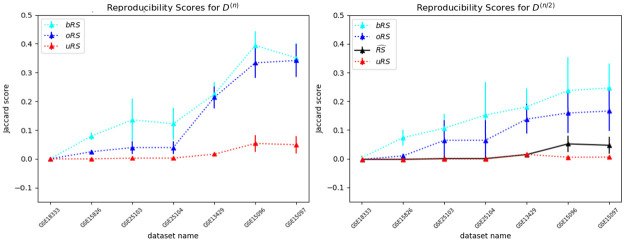
Empirical results for SNP datasets. Reproducibility scores (mean and standard deviation) for 7 SNP datasets, both for complete datasets with *n* subjects *D*^(*n*)^ (left) and for half-sized with $\frac{n}{2}$ subjects *D*^(*n*/2)^ (right), for 50 iterations. The x-axes (for both plots) is sorted by the value of the overbound oRS for the *D*^(*n*)^ datasets. We see, in both, that the over-bound oRS is consistently higher than the under-bound uRS. Moreover, the right plot shows that the “truth” $\hat{\text{RS}}$ is also within the range of oRS and uRS. (The bRS lines in the plots are based on the Bootstrap Overbound method; see Section C.1 in S1 Appendix).

Finally, similar to that experiment over the 18 continuous datasets, we examined the 7 discrete datasets and produced the reproducibility scores. Fig 8[left] shows the scores for each of the 7 SNP datasets, demonstrating that oRS ≥ uRS holds for the discrete cases as well. Fig 8[right] shows the scores for *D*^(*n*/2)^ datasets, and performs the same verification. Those figures also allow us to see the Jaccard scores (U1 above) range from essentially 0 to around 0.475 for the *D*^(*n*/2)^ datasets. In addition to the plots, Tables 1 and 2 present the relevant values.

**Table 2:** Results for all datasets when using half of the subjects–*i.e.*, *D*^(*n*/2)^. Reproducibility Scores and average number of biomarkers are shown in the form of mean ± standard deviation. (The caption for Table 1 describes the row ordering).

Name	Average #biomarkers	uRS %	$\hat{RS}$ %	oRS %
GDS968	1593.67 ± 173.35	26.0 ± 5.87	47.5 ± 3.95	48.6 ± 5.21
GSE7390*	1.38 ± 3.01	0 ± 0	0 ± 0	1.84 ± 2.1
GSE2034*	41.05 ± 144.8	0 ± 0	0 ± 0	6.39 ± 4.72
GSE1456*	58.38 ± 138.11	0 ± 0	0 ± 0	4.5 ± 4.41
GSE11121*	181.8 ± 265.5	0 ± 0	0.09 ± 0.2	9.48 ± 7.67
GDS2546	1266.61 ± 354.72	5.01 ± 7.15	30.8 ± 4.82	33.5 ± 6.3
GDS2545	2051.58 ± 494.77	6.38 ± 7.33	34.0 ± 4.09	38.1 ± 5.92
GDS2547	648.55 ± 255.09	1.82 ± 4.24	23.7 ± 5.86	27.3 ± 6.63
KIPAN	694.48 ± 398.88	0.88 ± 2	12.3 ± 4.91	19.3 ± 8.33
BRCA	36.75 ± 171.15	0 ± 0	0 ± 0	2.6 ± 4.2
GDS2771	457.17 ± 863.64	0.09 ± 0.62	0.32 ± 0.64	7.23 ± 8.48
GDS3966	2976.15 ± 811.92	10.6 ± 7.83	31.7 ± 4.71	37.9 ± 6.44
GDS4185	2.92 ± 40.20	0 ± 0	0 ± 0	0.701 ± 3.11
Metabric	1301.9 ± 504.80	1.97 ± 3.86	18.5 ± 3.86	21.6 ± 7.3
GDS4431	43.35 ± 284.56	0 ± 0	0 ± 0	2.55 ± 3.82
GDS4719	0.36 ± 1.88	0 ± 0	0 ± 0	2.8 ± 5.31
GDS2737	0.79 ± 7.33	0 ± 0	0 ± 0	1.73 ± 2.82
GDS5218	4207.29 ± 1589.79	7.96 ± 4.08	24.0 ± 5.09	31.5 ± 6.15
GSE13429	339.29 ± 170.11	1.73 ± 0.44	1.66 ± 0.44	14 ± 5.04
GSE25103	309.61 ± 78.24	0.13 ± 0.11	0.27 ± 0.14	6.63 ± 6.79
GSE15097	22788.62 ± 23342.37	0.77 ± 0.62	4.9 ± 2.99	16.8 ± 6.85
GSE15096	22393.87 ± 20624.09	0.74 ± 0.60	5.37 ± 2.9	16.1 ± 6.91
GSE25104	309.93 ± 78.14	0.13 ± 0.11	0.27 ± 0.14	6.64 ± 6.8
GSE15826	1.21 ± 5.41	0 ± 0	2.03 ± 0.06	1.21 ± 0.79
GSE18333	0.01 ± 0.1	0 ± 0	0 ± 0	0 ± 0

Section C in S1 Appendix provides the results of many additional empirical studies, showing how the reproduciblity scores change based on which (if any) MCC method is used, the specific *p*-value used for the t-test, the number of draws *k* used by the various approximations, and the size of the dataset *n*.

## 4 Discussion

### 4.1 Recommended use

As suggested above, whenever researchers have identified a possible set of biomarkers from a dataset *D* and Biomarker Discovery Algorithm BD, we encourage them to apply our analytic technique, and where appropriate, even our specific bounding algorithms (from https://biomarker.shinyapps.io/BiomarkerReprod/), to estimate the Reproducibility Score of this proposed set. If those scores (especially the lower bound uRS(*D*, BD)) are sufficiently high, they can use those biomarkers, confident that they (as a set) are reproducible. But if not, the researchers may want to explore other ways to identify reproducible biomarkers. One obvious approach is to use a larger dataset, with more instances, as we know that RS increases with the size of the dataset |*D*|; see Heuristic 7 and the analysis in Section D.2 in S1 Appendix. Alternatively (or in addition), recall the reproducibility depends on both the dataset, and the *Biomarker Discovery Algorithm* BD; perhaps some other BD_*test*,*p* − *val*, *MCC*_ would work better here? We could consider modifying all 3 of the components:

- While the MCC methods are designed to reduce the false positives, it is not clear whether they improve RS. Indeed, our experiments (Fig C.1 in S1 Appendix) show that RS values reduce with MCC—which points to using BD_*test*,*p*, *None*_.
- The non-reproducibility problem might be due to the statistical test used. As noted earlier, the t-test implicitly assumes a Gaussian distribution of the features (or the normality of residuals); perhaps another test would work better for some dataset / distribution of instances.
- Finally, they may want to modify the *p*-value, *p*, as other values of *p* may lead to better reproducibility.

### 4.2 Limitations

This paper provides an effective way to estimate the reproducibility of the biomarkers found from a dataset, using a biomarker discovery algorithm. While the message of this paper is very general, the specific analyses here all used the standard discovery algorithm BD_*t*, 0.05, *BH*_, to illustrate that the issues apply to the approaches commonly used. Section C in S1 Appendix explores some other discovery methods, that are also based on t-tests. While this use of t-tests implicitly assumes normality of the feature values, nothing in our general approach relies on this specific test—we could use other tests that make other parametric assumptions. (Note that our analysis appears to work effectively even when dealing with some Bernoulli (non-normal) features.) We anticipate the same approach would hold for other tests, such as Mann-Whitney, Wilcoxon or even multivariate analysis–but this is future work. All of our empirical studies dealt with standard datasets, whose outcomes are binary and whose values were either all real values or all categorical values; none had some of each. Our analytic model considers the overlap of biomarkers found from two datasets, of the same size—*e.g.*, we do not consider how the biomarkers obtained from a size-100 dataset, overlap with those from a size-300 dataset. Finally, the recommendations of the previous subsection suggest another future direction: produce the “BD′(*D*, *s*) algorithm” that, given a dataset *D* and a minimum score *s* > 0, returns the parameters [*test*, p-val, *MCC*] for a biomarker discovery algorithm BD_*test*,*p* − *val*, *MCC*_ that would produce a biomarker set whose Reproducibility Score would be at least *s*–*i.e.*, we expect that RS(*D*, BD_*test*,*p* − *val*, *MCC*_(⋅)) ≥ *s*, suggesting this level of reproducibility for the proposed biomarkers BD_*test*,*p* − *val*, *MCC*_(*D*).

### 4.3 Contributions

This paper has several contributions: (1) It motivates, then provides, a formal definition of reproducibility, that can help researchers evaluate a set of purported biomarkers; (2) It provides a pair of algorithms that can accurately bound this “reproducibility score” for a given dataset and biomarker discovery algorithm; (3) It provides empirical evaluation of these algorithms, over 25 different real-world datasets, to demonstrate that they work effectively; and (4) It introduces a freely-available website https://biomarker.shinyapps.io/BiomarkerReprod/ that runs these algorithms on the dataset entered by a user, and biomarker discovery system, which will allow users to easily evaluate the quality of the biomarker set produced. Given how easy it is to use this tool, we hope that future researchers will automatically use it to quickly evaluate the quality of the purported biomarkers, then include these estimates when they publish their biomarkers. We anticipate this analysis may also lead to new biomarker discovery algorithms, to optimize this reproducibility score.

## Supporting information

S1 Appendix (PDF)
