# Supplementary material for: Analyzing biomarker discovery: Estimating the reproducibility of biomarker sets
Source: PLoS One. 2022 Jul 28;17(7):e0252697. doi: 10.1371/journal.pone.0252697 (PMC9333302; doi:10.1371/journal.pone.0252697)
Supplement: S1 Appendix — (PDF) [file pone.0252697.s001.pdf]

Appendix

A Glossary

Term	Description
$\mathrm{BD}_{t,p,\chi}(D)$	**B**iomarker **D**iscovery algorithm: given a labeled dataset D, return the set of features (associated with D), that exhibit a class difference; see Eq 1. Here, the t in the subscript refers to the 2-sided t-test, the p for the p-value used, and χ to the MCC correction. Our canonical example is $\mathrm{BD}_{t,0.05,BH}(\,D\,)$, with p =0.05, and χ =BH corresponding to the Benjamini/Hochberg correction. We use $\mathrm{BD}(D)$ for a generic algorithm.
biomarker	"a characteristic (feature) that is objectively measured and evaluated as an indicator of normal biological processes, pathogenic processes, or pharmacologic responses to a therapeutic intervention" [1]
D, $D^{(n/2)}$	D refers to a labeled dataset, with n subjects, each described by r features, and an outcome. $D^{(n/2)}$ refers to a dataset formed containing half of the elements of the original dataset D – this is balanced, containing $n_+/2$ +-labeled subjects and $n_-/2$ −-labeled subject
feature	sometimes called the "independent variables" or "covariates" – here, we assume they are all real valued
gene expression	continuous value that indicates how much RNA of a specific gene has been expressed
GWAS	genome-wide association study
$J(\,A,\ B\,)$	Jaccard score of pair of sets A and B; see Eq 3
MCC	Multiple Comparison Corrections – here used as a generic term, which includes False Discovery Rate (FDR) correction, Family-Wise Error (FWE) Correction, etc.
n, $[n_+, n_-]$	By convention, n is the number of subjects in the dataset, which includes n_+ subjects that are labeled +, and n_- subjects labeled −.
$\mathrm{oRS}(\,D,\ \mathrm{BD}(\cdot),\ k\,)$	(an estimate of) an overbound of $\mathrm{RS}(\,D, \mathrm{BD}(\cdot)\,)$ where k = #repetitions; see Eq 6
outcome	sometimes called the "label", "response" or "dependent variable" – here, just considering binary (think "case vs control")
$\vec{p}(\cdot)$ $\vec{p}^{[n_+,n_-]}(\cdot)$	$\vec{p}(\cdot) = [\,p_{j,c}(\cdot)\,]_{j,c}$ is the $2 \times r$ matrix of the distributions for the 2 outcomes and r features; $\vec{p}^{[n_+,n_-]}(\cdot)$ is the distribution that draws, for each feature j, n_+ subjects from $p_{j,+}$ and n_- from $p_{j,-}$
$\widehat{p_D}(\cdot)$	empirical estimate of $\vec{p}(\cdot)$, based on the context of the dataset D
$\mathrm{RS}(\,D, \mathrm{BD}(\cdot)\,)$	"Reproducibility Score" of the biomarkers produced, by running biomarker-discovery algorithm $\mathrm{BD}(\cdot)$ on the labeled dataset D; see Eq 4
$\mathrm{RS}^*(\,\vec{p}(\cdot),\ [n_+, n_-],\ \mathrm{BD}(\cdot)\,)$	reproducibility score for the biomarkers produced by the $\mathrm{BD}(\cdot)$ biomarker discovery tool on two datasets of size $[n_+, n_-]$ drawn from $\vec{p}(\cdot)$; see Eq 2
SNP	single nucleotide polymorphism – discrete, with values {AA, Ab, bb} indicating homozygous dominant, heterozygous, and homozygous minor, respectively
subject	a "record" in the dataset (*e.g.*, a row in the table shown in Fig 1). Also called "element" or "instance"
$\mathrm{uRS}(\,D,\ \mathrm{BD}(\cdot),\ k\,)$	(an estimate of) an underbound of $\mathrm{RS}(\,D, \mathrm{BD}(\cdot)\,)$, where k = #repetitions; see Eq 10

B Notes from Text

This appendix provides various short notes related to material in the main text:

B.1 How biomarker discovery differs from supervised machine learning

We earlier noted a few differences between association studies versus predictive studies. Here, we list two others:

(a) In general, a predictive model provides some information about *an individual* (corresponding to a **row** of the matrix at the top of Fig 1) – *e.g.*, whether she has some specific disease. By contrast, an association study identifies *features* (each corresponding to a **column**), with the prediction that each considered a biomarker will exhibit some *population difference with respect to a dataset of many individuals.*

(b) It is relatively easy to evaluate the quality of a learned predictive model, by running that predictor on a held-out set of subjects. By contrast, there is no direct way to determine if a purported biomarker is correct. This is why we, instead, look for "consistency" of a set of biomarker discovery tools. That is, we hope that these discovered feature sets have low variance. (Note that they can have high-bias – *e.g.*, if they all set $p = 1$, then each discoverer will return all features; this will have low variance, but presumably high bias.)

B.2 Why do many papers not provide biological validation?

This may be because such biological validation is not yet implementable, or the technology is not yet available. Alternatively, the biological validation may be possible but be a major project that those authors hope to explore in future works.

B.3 Different notions of "Reproducibility"

There are (at least) be two sources of non-reproducibility in computational situations:

1. based on issues related to the specific code that is being run (*e.g.*, based on its many parameters and protocols), and

2. based on different datasets (drawn from the same distribution).

Here, however, we do *not* have to consider Issue#1, as our "Reproducibility Score" is defined for a given dataset D and also **a specific BiomarkerDiscovery algorithm** $\text{BD}(\cdot)$ **that is fixed and unambiguous**; see Section 2.2, etc.

Our analysis focuses only on Issue#2: dealing with different samples. This is the standard notion of scientific inquiry: researchers are seeking properties that are repeatable, for *any* appropriate dataset, which we view as a draw from a fixed (if initially unknown) underlying distribution. Note that this is not just the norm in science in general (*cf.*, `https://en.wikipedia.org/wiki/Reproducibility`), this is exactly what many many researchers are doing when seeking biomarkers, as this is the (implicit) basis of all biomarker association studies.

B.4 Our model (Eq 1) deals with just a single step

Some modern GWAStudies involve many phases – typically using one phase to reduce $\approx 10^6$ features to a few thousand based on one dataset, and then using a second dataset to reduce those features to a sub-subset, etc [2]. Here, our analysis is relevant to any one of these phases; see Fig 1. Also, some studies regress out covariates before finding biomarkers; here, we assume that this has happened and our analysis uses those already-regressed-out values.

B.5 Some features may only be important in combination

Sometimes a feature may be completely independent of the outcome, by itself, but become relevant, in combination with another (independent) feature. As an example, consider babies *in utero*, whose descriptions each include the Rh blood type of its mother, MRh $\in \{+, -\}$, and also of its father FRh $\in \{+, -\}$, among other features, where the outcome is the health H of that baby. Note that H may be completely uncorrelated with MRh, and also completely uncorrelated with FRh – which means neither MRh nor FRh could be a biomarker. However, suppose finding these blood factors are different MHr$\neq$FHr, increases the baby's risk. Assuming balanced sampling (with an equal number of MRh=+ and MRh=− subjects, and similarly for FRh), this means an effective predictive model would need to include both features, even though neither is a biomarker.

Moreover, we typically assume that a feature either increases the risk of a disease in all situations, or always decreases that risk. This in-utero-baby example shows this is not always the case: We see that MRh=+ can sometimes increase the risk (when FRh=−), and other times, decrease the risk (when FRh=+). Hence, a simple linear combination of feature values might not always be appropriate.

While this is an extreme situation – where each feature is completely irrelevant by itself – it is relatively common for a disease to be associated with many minor features; here again, it is possible that none of the features, by itself, shows sufficient class distinction

This also happens when the class is inherently heterogeneous – *e.g.*, "headache" can be based on various phenomena, including ischemic stroke, dehydration, migraine, etc., each with various different factors. This is believed to happen with essentially all complex genetic disorders, especially when underlying pathologies are not known.

These situations argue that a *panel* of features can sometimes be more appropriate than individual features. If the model starts with a pre-defined combination of a set of features – *e.g.*, a simple average of a specific set of gene expression values, or a set of heterozygous settings in a specific set of SNPs – then we can view that combination as a (super-)feature, and let it be a column in the matrix of Fig 1; the analysis described in the paper still apply. Note, however, that here we assume this super-feature construction is known initially, and in particular, this paper is *not* exploring ways to find these features – *i.e.*, it is not describing machine learning tools for producing new super-features. We are also not considering multivariate approaches, where the relevance of one feature is implicitly conditioned on other features simultaneously – *e.g.*, multiple regression models.

C Exploring Other Settings

For consistency, all of the experiments in the main text used the same $\text{BD}_{t,0.05,BH}$ biomarker discovery algorithm. However, there are many other approaches that have been used in other association studies. Here, we continue to consider only the t-test as the main statistical significance test. First, Appendix C.1 explores the obvious bootstrap method, and demonstrates that its estimate is consistently worse than our over-bound method. The other sub-appendices consider only the algorithms explicitly described in the main text. Appendix C.2 explores different options for the p-value threshold and the p-value adjustment method, to see how changing these affect the reproducibility results. This paper introduced two different approximations for the *Reproducibility Score* – uRS and oRS. Appendix C.3 explores how these approximations change as we adjust the size of the dataset n, and the number of iterations of running the algorithms, k. Finally, Appendix C.4 motivates then presents the PO score, an alternative to the Jaccard score.

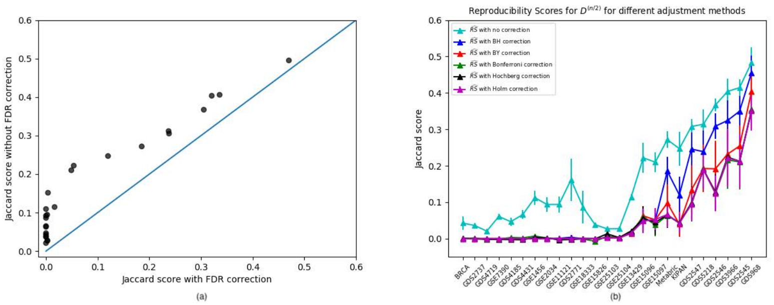

Fig C.1. Reproducibility Scores with different correction methods. (a) Scatter plot of Reproducibility Scores for all 25 datasets: each (x, y) point represents the average $D^{(n/2)}$ Jaccard scores for a single dataset (using disjoint subset pairs), where the x-value represents the score with MCC correction ($\text{BD}_{t,0.05,BH}$) and the y-value which represents the score without MCC correction ($\text{BD}_{t,0.05,-}$). Each point above the diagonal line means the MCC correction led to inferior performance for the associated dataset. (b) Reproducibility scores $\widehat{\text{RS}}$ for all 25 datasets when using $D^{(n/2)}$, for different p-value adjustment methods – *i.e.*, $\text{BD}_{t,0.05,\chi}$ for 5 different FDR adjustment methods χ, including BH and “no”.

C.1 Bootstrap Sampling Method

We initially explored the standard bootstrap sampling method bRS as an overbound measure. Given a dataset D, this bRS method produces new datasets by drawing $n = |D|$ subjects from D, with replacement: In particular, for $i = 1..k$, bRS(D, BD($\cdot$), k) draws a pairs of such bootstrap datasets $D_{i,1}$ and $D_{i,2}$ from D, then computes the Jaccard score of their respective BD($\cdot$)-biomarkers, and returns the average. Figs 7 and 8 show these results for continuous and SNP datasets, respectively. These experiments show, for all 25 datasets, and for both $D^{(n)}$ and $D^{(n/2)}$ datasets (when using all n, and also when using $\frac{n}{2}$ subjects), that this method is a more extreme over-bound:

$$\text{bRS}(\,D,\,\text{BD}(\cdot),\,k\,) \quad\geq\quad \text{oRS}(\,D,\,\text{BD}(\cdot),\;k\,) \quad\geq\quad \text{RS}(\,D,\,\text{BD}(\cdot)\,) \tag{14}$$

which means it is a less-useful measure.

C.2 p-value adjustment methods and p-value threshold

Fig C.1(a) shows the effect of Benjamini and Hochberg (BH) MCC correction on the reproducibility scores, by comparing the average Jaccard score for the biomarkers found for a pair of complementary $D^{(n/2)}$ datasets when using the standard MCC correction ($\text{BD}_{t,0.05,BH}$), versus without MCC correction ($\text{BD}_{t,0.05,-}$) across all datasets. We see that this MCC correction is *detrimental*, as it reduces the reproducibility scores across all datasets: While it is designed to reduce false discoveries (and hence increase precision), this may mean it is reducing recall, which collectively leads to a smaller Jaccard score.

There are many other methods for reducing MCC, in addition to Benjamini+Hochberg (BH) [3], including: Benjamini and Yekutieli (BY) [4], Bonferroni [5], Hochberg [6] and Holm [7]. Fig C.1(b) shows the results of these 5 MCC methods, as well as the "no-FDR" approach, over all 25 datasets – here showing RS with respect to the half-datasets $D^{(n/2)}$; see Eq 8. We see again that "no-MCC" remains the best approach, and that BH is the 2nd best, followed by the others.

We also anticipate the RS score will depend on the p-value threshold used to determine the significance of each feature – *i.e.*, $\text{BD}_{t,\tau,BH}$, for various $\tau \in (0, 0.1)$. While most studies use a threshold of $\tau = 0.05$, this number is fairly arbitrary. Here we explored how the RS changed with different values of τ. Fig C.2 shows that the reproducibility score for $D^{(n/2)}$ appears monotonic with τ – within this $\tau \in (0, 0.1)$ range, larger τ produces higher RS.

C.3 Changing k, the number of data-subset pairs drawn

Each of our approximation algorithms uses k, the number of data-subset pairs drawn. As we often work with large datasets, these algorithms can be very time consuming (even though they have been optimized), motivating us to explore how these algorithms scale, based on this parameter.

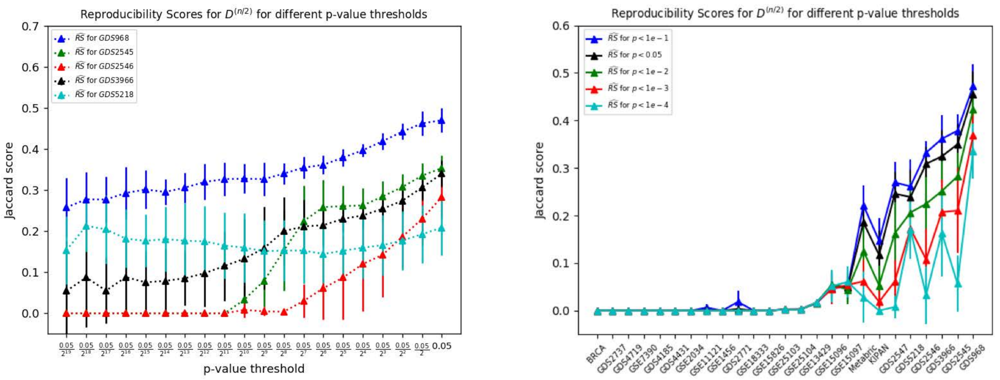

Fig C.2. Reproducibility Scores using different p-value thresholds. Reproducibility scores $\widehat{\mathrm{RS}}$ for various datasets when using $D^{(n/2)}$ for different p-value thresholds – $\mathrm{BD}_{t,\tau,BH}$, for various $\tau \in (0, 0.1)$. (left) considers 5 datasets, for a range of 20 different values of τ; (right) plots the Jaccard scores for all 25 datasets, for 5 different values of τ.

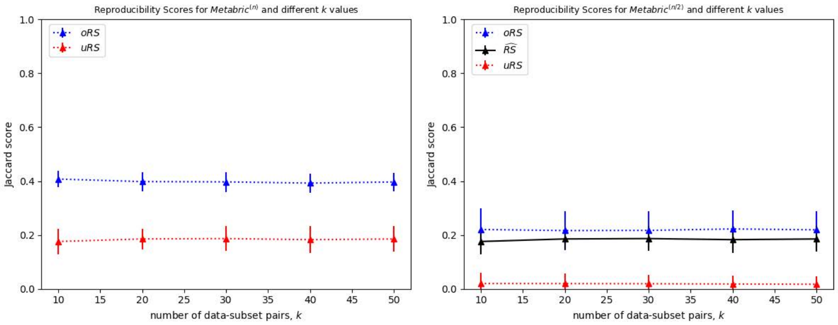

Fig C.3. Reproducibility scores for different numbers of iterations, for the Metabric$^{(n/2)}$ datasets.

We therefore ran these algorithms for our largest dataset, Metabric, but varied this k. Fig C.3 shows that we obtained very similar results, whether we used $k = 10$, up to $k = 50$, for both uRS and oRS, for both Metabric and Metabric$^{(n/2)}$. We also computed the mRS values for all 25 datasets D when running the algorithm for $k = 10$ versus $k = 50$, and found the difference between the two, $\text{mRS}(\, D^{(n/2)}), \text{BD}(\cdot), \text{k=50}\,) -$ $\text{mRS}(\, D^{(n/2)}, \text{BD}(\cdot), \text{k=10}\,)$, is very close to 0 for most cases; see Fig C.4.

C.4 PO Score

We have used Jaccard score as our similarity measure throughout all of our experiments. This is a symmetric measure, meaning it provides information about a pair of datasets $\{\ A,\ B\ \}$ where $J(\ A,\ B\ ) = J(\ B,\ A\ )$, which can be very useful when comparing the results from different experiments or evaluating the outcome when trying to replicate results from a previous study.

However, there are other options for the similarity measure that are not symmetric and can be provided together with the set of biomarkers for each dataset. One of these options is the PO score, which is used by the Zou *et al.* [8] meta-study: for each (ordered) pair of biomarker sets $[B_i, B_j]$,

$$PO(\, B_i,\, B_j\,) \quad = \quad \frac{|B_i \cap B_j|}{|B_i|} \times 100\% \;. \tag{15}$$

Notice this is an asymmetric variant of the Jaccard score (Eq 3). (As that paper also reported the number of biomarkers found for each dataset, we could therefore recover the associated Jaccard score.) This measure can be reported with a set of biomarkers, to estimate the number of these biomarkers that should recur, if performing another similar study.

D Arguments Supporting the Heuristics

The overbound and underbound algorithms, uRS and oRS, are based on some intuitions, appearing as Heuristics 7 and 9. Our empirical results, Table 1 and 2, support these claims over 25 real-world datasets – as uRS is consistently below $\widehat{RS}$, which is below oRS (as visualized by the relative heights of uRS vs $\widehat{RS}$ vs oRS in Figs 4(a,b)).

This appendix further motivates these heuristics. Subappendices D.1 and D.2 first provide arguments that motivate these heuristics (both focus on simple use of t-test $\text{BD}_{t,\alpha,-}$), then Appendix D.3 provides further empirical evidence that these claims hold in practice. Appendix D.4 provides additional theoretical justifications for the overlaps claims underlying Heuristic 7.

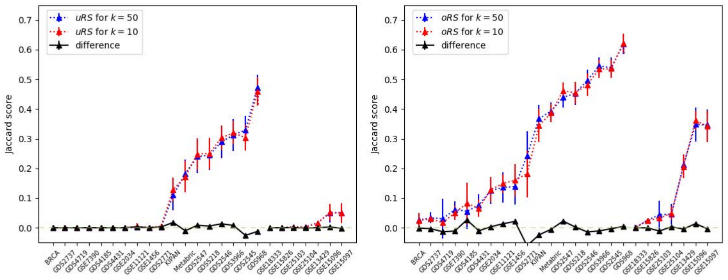

Fig C.4. uRS **and** oRS **values for all 25 datasets when running** $k = 10$ **versus** $k = 50$ **iterations.** Note the black line, hovering around 0, is the difference between the uRS (left) values when using $k = 10$ iterations and when using $k = 50$ iterations; and similarly for the oRS values (right).

D.1 Motivation for Heuristic 7

Here, we motivate Heuristic 7 by explaining why we expect the Jaccard score of the biomarkers found from two related datasets, to increase as the subjects forming the dataset have larger overlap. We initially consider a single feature, call it g, and explore a relevant statistic from these two datasets. Here, we assume that every subject of g associated with a positively (resp., negatively) labeled subject is drawn from a distribution with mean μ_+ and variance σ_+^2 (resp., μ_- and σ_-^2). (Of course, for non-biomarkers, $\mu_+ = \mu_0$ and $\sigma_+^2 = \sigma_-^2$.) Each dataset has n^+ positive subjects and n^- negative subjects, and these are drawn i.i.d., except that each dataset includes r^+ common positive subjects and r^- common negative subjects.

For notation, let $D_1 = E_1^+ \cup E_1^-$ and $D_2 = E_2^+ \cup E_2^-$ be the two datasets where E_1^+ and E_2^+ (resp., E_1^- and E_2^-) are the values of feature g associated with the positively (resp., negatively) labeled subjects:

$$\begin{aligned} E_1^+ &= \{a_1, \ldots, a_{r^+}, \quad b_1, b_2, \ldots, b_{k^+}\} \\ E_2^+ &= \{a_1, \ldots, a_{r^+}, \quad c_1, c_2, \ldots, c_{k^+}\} \\ E_1^- &= \{d_1, \ldots, d_{r^-}, \quad e_1, e_2, \ldots, e_{k^-}\} \\ E_2^- &= \{d_1, \ldots, d_{r^-}, \quad f_1, f_2, \ldots, f_{k^-}\} \end{aligned}$$

where each a_i, b_i, c_i is drawn, i.i.d., from a distribution with variance σ_+^2, and each d_i, e_i, f_i is drawn, i.i.d., from a distribution with variance σ_-^2.

As desired, E_1^+ and E_2^+ share $r^+ = n^+ - k^+$ elements in common, and E_1^- and E_2^- share $r^- = n^- - k^-$ elements in common.

Now define the means:

$$\begin{aligned} \bar{X}_1^+ &= \tfrac{1}{n^+}\left[\textstyle\sum_{i=1}^{r^+} a_i + \sum_{j=1}^{k^+} b_j\right] & \bar{X}_1^- &= \tfrac{1}{n^-}\left[\textstyle\sum_{i=1}^{r^-} d_i + \sum_{j=1}^{k^-} e_j\right] \\ \bar{X}_2^+ &= \tfrac{1}{n^+}\left[\textstyle\sum_{i=1}^{r^+} a_i + \sum_{j=1}^{k^+} c_j\right] & \bar{X}_2^- &= \tfrac{1}{n^-}\left[\textstyle\sum_{i=1}^{r^-} d_i + \sum_{j=1}^{k^-} f_j\right] \end{aligned}$$

then define the joint total variance values as

$$\begin{aligned} \bar{S}_1 &= \sum_{i=1}^{r^+}(a_i - \bar{X}_1^+)^2 + \sum_{j=1}^{k^+}(b_i - \bar{X}_1^+)^2 + \sum_{i=1}^{r^-}(d_i - \bar{X}_1^-)^2 + \sum_{j=1}^{k^-}(e_i - \bar{X}_1^-)^2 \\ \bar{S}_2 &= \sum_{i=1}^{r^+}(a_i - \bar{X}_2^+)^2 + \sum_{j=1}^{k^+}(c_i - \bar{X}_2^+)^2 + \sum_{i=1}^{r^-}(d_i - \bar{X}_2^-)^2 + \sum_{j=1}^{k^-}(f_i - \bar{X}_2^-)^2 \end{aligned}$$

Note that $\bar{S}_1$ and $\bar{S}_2$ should be extremely similar, as each is the sum of n^+ terms, each with expected value of σ_+^2, and of n^- terms each with expected value σ_-^2. We will assume they are effectively the same value; call it $\bar{S}_{1,2} = \frac{\bar{S}_1 + \bar{S}_2}{2}$.

Now observe the t-statistics of each dataset:

$$\mathrm{t}(E_1) = \frac{\bar{X}_1^+ - \bar{X}_1^-}{\kappa\sqrt{\bar{S}_1}} \qquad \mathrm{t}(E_2) = \frac{\bar{X}_2^+ - \bar{X}_2^-}{\kappa\sqrt{\bar{S}_2}} \tag{16}$$

where $\kappa = \sqrt{\frac{1}{n_+ + n_- - 2}\left[\frac{1}{n_+} + \frac{1}{n_-}\right]}$, and define

$$Q \quad = \quad \mathrm{t}(\,E_1\,) - \mathrm{t}(\,E_2\,) \quad = \quad \frac{1}{\kappa}\left[\frac{\bar{X}_1^+ - \bar{X}_1^-}{\sqrt{\bar{S}_1}} \;-\; \frac{\bar{X}_2^+ - \bar{X}_2^-}{\sqrt{\bar{S}_2}}\right] \quad \approx \quad \frac{1}{\kappa}\left[\frac{(\bar{X}_1^+ - \bar{X}_1^-) \;-\; (\bar{X}_2^+ - \bar{X}_2^-)}{\sqrt{\bar{S}_{1,2}}}\right]$$

as the different between the t-statistics of these datasets. Note that the expected value $E[Q] = 0$. We want to show that its variance decreases as r^+ or r^- increases.

We will view $\bar{S}_{1,2}$ as a constant, and focus on the numerator

$$\begin{aligned} R \quad &= \quad (\bar{X}_1^+ - \bar{X}_1^-) \;-\; (\bar{X}_2^+ - \bar{X}_2^-) \quad = \quad (\bar{X}_1^+ - \bar{X}_2^-) \;-\; (\bar{X}_2^+ - \bar{X}_1^-) \\ &= \quad \frac{1}{n^+}\sum_{j=1}^{k^+}(b_j - c_j) \quad + \quad \frac{1}{n^-}\sum_{j=1}^{k^-}(f_j - e_j) \end{aligned}$$

We can now observe that

$$\begin{aligned} \mathrm{Var}\left[\,R \mid k^+, k^-\,\right] \quad &= \quad \sum_{j=1}^{k^+}\mathrm{Var}\left[\frac{1}{n^+}b_j\right] + \mathrm{Var}\left[\frac{1}{n^+}c_j\right] \quad + \quad \sum_{j=1}^{k^-}\mathrm{Var}\left[\frac{1}{n^+}f_j\right] + \mathrm{Var}\left[\frac{2}{n^+}e_j\right] \\ &= \quad \sum_{j=1}^{k^+}(\frac{1}{(n^+)^2}\sigma_+^2 + \frac{1}{(n^+)^2}\sigma_+^2 \quad + \quad \sum_{j=1}^{k^-}\frac{1}{(n^-)^2}\sigma_-^2 + \frac{1}{(n^-)^2}\sigma_-^2 \qquad (17) \\ &= \quad \frac{2k^+}{(n^+)^2}\sigma_+^2 \quad + \quad \frac{2k^-}{(n^-)^2}\sigma_-^2 \qquad (18) \end{aligned}$$

As a sanity check: if $k^+ = k^- = 0$, then E_1 and E_2 are identical, meaning that they will have the same t-statistics, which means Q and hence R will be 0, as confirmed by this formula. Clearly this quantity **decreases** as k^+ and k^- increases, which means, as $r^+ = n^+ - k^+$ and $r^- = n^- - k^-$ **increase**, we expect E_1 and E_2 to agree more with respect to the biomarker g – either both agree it will be a biomarker, or both agree it will not.

Let $\mathrm{BD}(E_1[r])$, (resp., $\mathrm{BD}(E_1[r])$) be the set of biomarkers found (by our $\mathrm{BD}(\cdot)$) for E_1 (resp E_2), when there are $r = [r^+, r^-]$ common elements, and let its complement $n\,\mathrm{BD}(E_1[r]) \;=\; F - \mathrm{BD}(E_1[r])$ and $n\,\mathrm{BD}(E_2[r]) \;=\; F - \mathrm{BD}(E_2[r])$ be the set of non-biomarkers, using F as the set of all features. The argument above suggests that $\mid \mathrm{BD}(E_1[r]) \;\cap\; \mathrm{BD}(E_2[r]) \mid$ monotonically increases with r, as does $\mid n\,\mathrm{BD}(E_1[r]) \;\cap\; n\,\mathrm{BD}(E_2[r]) \mid$.

Now consider the Jaccard score, and notice

$$J(\,\mathrm{BD}(E_1[r]),\ \mathrm{BD}(E_2[r])\,) \quad = \quad \frac{\mid \mathrm{BD}(E_1[r]) \;\cap\; \mathrm{BD}(E_2[r]) \mid}{|F| \;-\; \mid n\,\mathrm{BD}(E_1[r]) \;\cap\; n\,\mathrm{BD}(E_2[r]) \mid} \qquad (19)$$

where $|\chi|$ is the total number of elements in the set χ. As r increases, we expect the numerator to increase, and the denominator to decrease, both of which means the ratio will increase.

(In general, we expect smaller values of $\mathrm{Var}\,[\,R\,]$ to increase the chance that E_1 and E_2 will agree on the status of g. There is one exception here: say E_2 thinks that g is a biomarker as $\mathrm{t}(\,E_2\,)$ is very negative, but E_1 thinks it is not a biomarker, as $\mathrm{t}(\,E_1\,)$ is near 0. Here, imagine a variant of E_1 – call it E_1' – had a larger value of $\mathrm{t}(\,E_1'\,)$; large enough that it this value meant that g was considered a biomarker. Here E_1 and E_2 disagreed about g with their $Q = \mathrm{t}(\,E_1\,) - \mathrm{t}(\,E_2\,)$, but E_1' and E_2 agreed about g, despite having a larger value of $Q' = \mathrm{t}(\,E_1'\,) - \mathrm{t}(\,E_2\,)$. This is very unlikely, as the numerator (and hence the sign) of $\mathrm{t}(\,E_1\,)$ is basically an empirical estimate of $\mu_1 - \mu_2$,

which means finding a large positive value for $\mathrm{t}(\,E_1\,)$ is unlikely unless $\mu_1 \gg \mu_2$ – but this condition means it is very unlikely that $\mathrm{t}(\,E_2\,)$ (which is another estimate of $\mu_1 - \mu_2$) will be very negative. That is, if τ if the threshold for declaring a feature to be a biomarker: $P(\mathrm{t}(\,E_2\,) < -\tau \,|\, \mu_1 \gg \mu_2)$ is tiny. Similarly,

$$P(\,\mathrm{t}(\,E_1\,) > \tau,\ \mathrm{t}(\,E_2\,) < -\tau \ \mid\ \mu_1 \gg \mu_2\,) \quad \ll \quad \epsilon \tag{20}$$

$$P(\,\mathrm{t}(\,E_1\,) > \tau,\ \mathrm{t}(\,E_2\,) < -\tau \ \mid\ \mu_1 \approx \mu_2\,) \quad \ll \quad \epsilon \tag{21}$$

$$P(\,\mathrm{t}(\,E_1\,) > \tau,\ \mathrm{t}(\,E_2\,) < -\tau \ \mid\ \mu_1 \ll \mu_2\,) \quad \ll \quad \epsilon \tag{22}$$

where "$\ll \epsilon$" means a very small probability. Eq 20 holds as $\mathrm{t}(\,E_2\,) < -\tau$ is unlikely in this case; Eq 21 as both $\mathrm{t}(\,E_2\,) < -\tau$ and $\mathrm{t}(\,E_1\,) > \tau$ are each unlikely in this case; and Eq 22 as $\mathrm{t}(\,E_1\,) > \tau$ is unlikely in this case.)

Final note: This derivation is for the general statement of Heuristic 7. Our specific oRS algorithm imposes an additional constraint – in essence, that E_1 will have two copies of each b_j and each e_j, and that E_2 will have two copies of each c_j and each f_j. The claim still holds – but with a variance for R that is twice as large as Eq 18: there are now only half as many b_js (and c_j, e_j, f_j), but each is implicitly multiplied by $\frac{2}{n^+}$ or $\frac{2}{n^-}$, and variance goes with the square of that constant.

D.2 Motivation for Heuristic 9

Reusing the notation from above, assume D_1 and D_2 are each samples of size n, drawn from n^+ positive subjects and n^- negative subjects, independently for all of the features F. We also use BM to denote the set of all true biomarkers and NBM to denote the set of all true non-biomarkers.

For each gene g, our use of simple independent t-test means there are probabilities p, $q(n)$ such that

$$P(\,g \in \mathrm{BD}_{t,p,no}(D) \,|\, g \in \textsc{nbm}\,) \quad = \quad p \tag{23}$$

$$P(\,g \in \mathrm{BD}_{t,p,no}(D) \,|\, g \in \textsc{bm}\,) \quad = \quad q(n) \tag{24}$$

(Recall that $n = n^+ + n^-$ is the total number of subjects; we will insist on a constant ratio of $n^+ : n^-$ as we increase the total number of subjects.) Eq 23 is the definition of false-positive, which corresponds to the p-value of the t-test; Eq 24 is the "power" of the statistical test; we only need to know that $q(n)$ is monotonically increasing with n.

Given $E_1[n]$ and $E_2[n]$ are each based on n draws from the $\vec{p}$ distribution, we define the expected value

$$EJ(\,E_1[n],\ E_2[n]\,) \quad = \quad \mathbb{E}\left(\frac{|\,\mathrm{BD}(E_1[n])\ \cap\ \mathrm{BD}(E_2[n])|}{|\,\mathrm{BD}(E_1[n])\ \cup\ \mathrm{BD}(E_2[n])|}\right) \tag{25}$$

Heuristic 9 is claiming that this function is monotonically increasing as function of n.

To motivate this claim, we instead approximate this expectation of a ratio EJ (Eq 25) by the ratio of the expectations, $\tilde{J}$ (Eq 26, below), which Lemma 1 proves is monotonically increasing in n.

Lemma 1 *Let E_1, E_2 each by drawn from $\vec{p}(\cdot)$ of size n – drawing $n_+ \in \mathbb{Z}^+$ instances from the joint distribution $[p_{1,1}(\cdot), \ldots, p_{r,1}(\cdot)]$ associated with the outcome of $+$-instances, and $n_- \in \mathbb{Z}^+$ instances from the joint distribution $[p_{1,2}(\cdot), \ldots, p_{r,2}(\cdot)]$ associated with the outcome of $-$-instances. Assuming that biomarkers are based on $\mathrm{BD}_{t,p,no}(D)$, whose true positive rate is $q = q(n)$, and let*

$$\tilde{J}(n; E_1,\ E_2) \quad = \quad \frac{\mathbb{E}(\ |\,\mathrm{BD}(E_1)\ \cap\ \mathrm{BD}(E_2)|\ )}{\mathbb{E}(\ |\,\mathrm{BD}(E_1)\ \cup\ \mathrm{BD}(E_2)|\ )} \tag{26}$$

Now assume that $q = q(n)$ is increasing in n, and that $q > p/2$. Then $\tilde{J}(\, n;\, E_1,\, E_2\,)$ increases as n increases.

Proof: We first consider a single feature g, and consider the likelihoods that the two sample E_1 and E_2 will agree:

1. What is the chance that E_1 and E_2 both agree that g is a biomarker?

(1a) If g is a biomarker:

$$\begin{aligned} P(\, g \in \mathrm{BD}(E_1),\; g \in \mathrm{BD}(E_2) \,|\, g \in \textsc{bm}\,) &= P(\, g \in \mathrm{BD}(E_1) \,|\, g \in \textsc{bm}\,) \;\times\; P(\, g \in \mathrm{BD}(E_2) \,|\, g \in \textsc{bm}\,) \\ &= q(n) \;\times\; q(n) \end{aligned}$$

as the draws leading to E_1 and E_2 are independent.

(1b) If g is not a biomarker:

$$\begin{aligned} P(\, g \in \mathrm{BD}(E_1),\; g \in \mathrm{BD}(E_2) \,|\, g \notin \textsc{bm}\,) &= P(\, g \in \mathrm{BD}(E_1) \,|\, g \notin \textsc{bm}\,) \;\times\; P(\, g \in \mathrm{BD}(E_2) \,|\, g \notin \textsc{bm}\,) \\ &= p \;\times\; p \end{aligned}$$

2. What is the chance that E_1 and E_2 both agree that g is *not* a biomarker? Here again use $n\,\mathrm{BD}(D) = F - \mathrm{BD}(D)$ as the set of *non-biomarkers* found by BD – using F to represent the set of all features. Here we again deal first with the "g is a biomarker", then "g is not a biomarker", cases:

$$\begin{aligned} P(\, g \notin \mathrm{BD}(E_1),\; g \notin \mathrm{BD}(E_2) \,|\, g \in \textsc{bm}\,) &= P(\, g \notin \mathrm{BD}(E_1) \,|\, g \in \textsc{bm}\,) \;\times\; P(\, g \notin \mathrm{BD}(E_2) \,|\, g \in \textsc{bm}\,) \\ &= (1 - q(n)) \;\times\; (1 - q(n)) \end{aligned}$$

$$\begin{aligned} P(\, g \in n\,\mathrm{BD}(E_1),\; g \in n\,\mathrm{BD}(E_2) \,|\, g \notin \textsc{bm}\,) &= P(\, g \in n\,\mathrm{BD}(E_1) \,|\, g \notin \textsc{bm}\,) \;\times\; P(\, g \in n\,\mathrm{BD}(E_2) \,|\, g \notin \textsc{bm}\,) \\ &= (1 - p) \;\times\; (1 - p) \end{aligned}$$

Hence, the expected size of the intersection of the biomarkers found by both samples (resp., NOT found by both) is

$$\begin{aligned} &\mathbb{E}[\; \mathrm{BD}(E_1) \;\cap\; \mathrm{BD}(E_2)\;] \\ &= \sum_{g \in \mathrm{BM}} P(\, g \in \mathrm{BD}(E_1),\; g \in \mathrm{BD}(E_2) \,|\, g \in \textsc{bm}\,) \;+\; \sum_{g \notin \mathrm{BM}} P(\, g \in \mathrm{BD}(E_1),\; g \in \mathrm{BD}(E_2) \,|\, g \notin \textsc{bm}\,) \\ &= |\textsc{bm}| \;\times\; p^2 \;+\; (|F| \;-\; |\textsc{bm}|) \;\times\; q(n)^2 \end{aligned}$$

$$\begin{aligned} &\mathbb{E}[\; n\,\mathrm{BD}(E_1) \;\cap n\,\mathrm{BD}(E_2)\;] \\ &= \sum_{g \in \mathrm{BM}} P(\, g \in n\,\mathrm{BD}(E_1),\; g \in n\,\mathrm{BD}(E_2) \,|\, g \in \textsc{bm}\,) \;+\; \sum_{g \notin \mathrm{BM}} P(\, g \in n\,\mathrm{BD}(E_1),\; g \in n\,\mathrm{BD}(E_2) \,|\, g \notin \textsc{bm}\,) \\ &= |\textsc{bm}| \times (1-p)^2 \;+\; (|F| \;-\; |\textsc{bm}|) \;\times\; (1 - q(n))^2 \end{aligned}$$

We now use the observation, from Eq 19, that $\mathrm{BD}(E_1) \;\cup\; \mathrm{BD}(E_2) \;=\; F \;-\; (n\,\mathrm{BD}(E_1) \;\cap\; n\,\mathrm{BD}(E_2)\;)$, which means Eq 26 reduces to

$$\begin{aligned} \tilde{J}(n; E_1,\; E_2) &= \frac{|\textsc{bm}| \;\times\; p^2 \;+\; (|F| \;-\; |\textsc{bm}|) \;\times\; q(n)^2}{|F| \;-\; (|\textsc{bm}| \times (1-p)^2 \;+\; (|F| \;-\; |\textsc{bm}|) \;\times\; (1 - q(n))^2)} \\ &= \frac{t \;\times\; p^2 \;+\; s \;\times\; q(n)^2}{1 \;-\; [t \;\times\; (1-p)^2 \;+\; s \;\times\; (1 - q(n))^2]} \end{aligned}$$

where $t = |\textsc{bm}|/|F|$ is the fraction of biomarkers (which is typically very small), and $s = 1 - t$ as the fraction of non-biomarkers. Also write $q = q(n)$.

Now note that $\frac{\partial \tilde{J}}{\partial n} \;=\; \frac{\partial \tilde{J}}{\partial q}\frac{\partial q}{\partial n}$, and recall that $\frac{\partial q}{\partial n} \geq 0$. Hence, to show that $\tilde{J}$ increases with n, it suffices to show that $\frac{\partial \tilde{J}}{\partial n} \geq 0$. First,

$$\frac{\partial \tilde{J}}{\partial q} = \frac{1 - \left[t(1-p)^2 - s(1-q)^2\right] \times 2sq - [tp^2 + sq^2] \times (-s) \times 2 \times (1-q) \times (-1)}{[1 - t(1-p)^2 - s(1-q)^2]^2}$$

As the denominator $[1 - t \times (1-p)^2 - s \times (1-q)^2]^2$ is positive, so we can ignore it. (Below we use $\propto$ to mean "same wrt sign"):

$$\begin{aligned}
\frac{\partial J}{\partial q} &\propto [1 - t(1-p)^2 - s(1-q)^2] \times 2s\,q - [tp^2 + sq^2] \times 2s \times (1-q) \\
&\propto [1 - t(1-p)^2 - s(1-q)^2] \times q + [tp^2 + sq^2] \times (q-1) && (27)\\
&= (q - qt(1+p^2-2p) - sq(1+q^2-2q)] + [qtp^2 + qsq^2] - [tp^2 + sq^2] \\
&= q - qt - qtp^2 + 2pqt - sq - sq^3 + 2sq^2 + qtp^2 + sq^3 - tp^2 - sq^2 \\
&= q - qt + 2pqt - sq + sq^2 - tp^2 \\
&= q - qt + 2pqt - (1-t)q + (1-t)q^2 - tp^2 \\
&= q - qt + 2pqt - q + tq + q^2 - tq^2 - tp^2 \\
&= q^2 - t(q^2 + p^2 - 2pq) \\
&= q^2 - t(q-p)^2 \\
&\geq q^2 - (q-p)^2 = p(2q-p) && (28)\\
&\propto q - \frac{p}{2} && (29)\\
&\geq 0 && (30)
\end{aligned}$$

where the "$\propto$" in Line 27 as $2s > 0$; the "$\geq$" in Line 28 follows from $t > 0$;, the "$\propto$" in Line 29 from knowing that p, and hence $p/2$, is > 0, and the "$\geq$" in Line 30 from our assumption that $q > \frac{p}{2}$.

■ (Lemma 1)

D.3 Empirical Evidence for Heuristics 7 and 9

Our empirical results, over 25 datasets, demonstrate that algorithms based on Heuristics 7 and 9 work effectively; see Tables 1 and 2. This subappendix provides further empirical evidence by running a number of simulations, based on realistic distributions of data. In particular, we form distributions based on each of 4 real-world datasets: $\Gamma =\{$ GDS968, GDS2771, GSE11121, GDS4185 $\}$. (We selected these datasets to span a wide range of situations – in terms of number of subjects, number of features and the Reproducibility Scores; see Fig 5.) For each dataset, we compute the empirical mean and variance of each feature j and for each outcome c, then define $p_{j,c}(\cdot)$ to be a univariate Gaussian with this mean and variance. We can then form new datasets by drawing $n_+(\rho)$ subjects of each $c = +$ outcome and $n_-(\rho)$ subjects of each $c = -$ outcome, matching the size of the original $\rho \in \Gamma$ dataset. (We will use $n(\rho)$ to be the total number of elements in that dataset.)

We then ran 3 experiments on each of the 4 distributions of data. The first experiment explored the claim that "Underbound $\leq$ True Jaccard $\leq$ Overbound". Here, we simulated 100 pairs of datasets, for each of the 4 real-world datasets, then computed the average true Jaccard score between the biomarker sets found for each pair. We also computed the average uRS and oRS score for each of these simulated datasets. Fig D.5 shows the histograms of values for each of these 4 different distributions. As expected, we see no overlap between the histograms of the underbound, true and overbound, except for GDS968. Here, we found that the true Jaccard was higher than the overbound in just 8 of the 100 cases; and the largest difference here was 0.013 (and the average difference, over these 8, was only 0.004). Fig D.6 shows the median and interquartile range for each of the 3 algorithms $\{$uRS, true_Jaccard, oRS$\}$ for each of the 4 distributions in Γ.

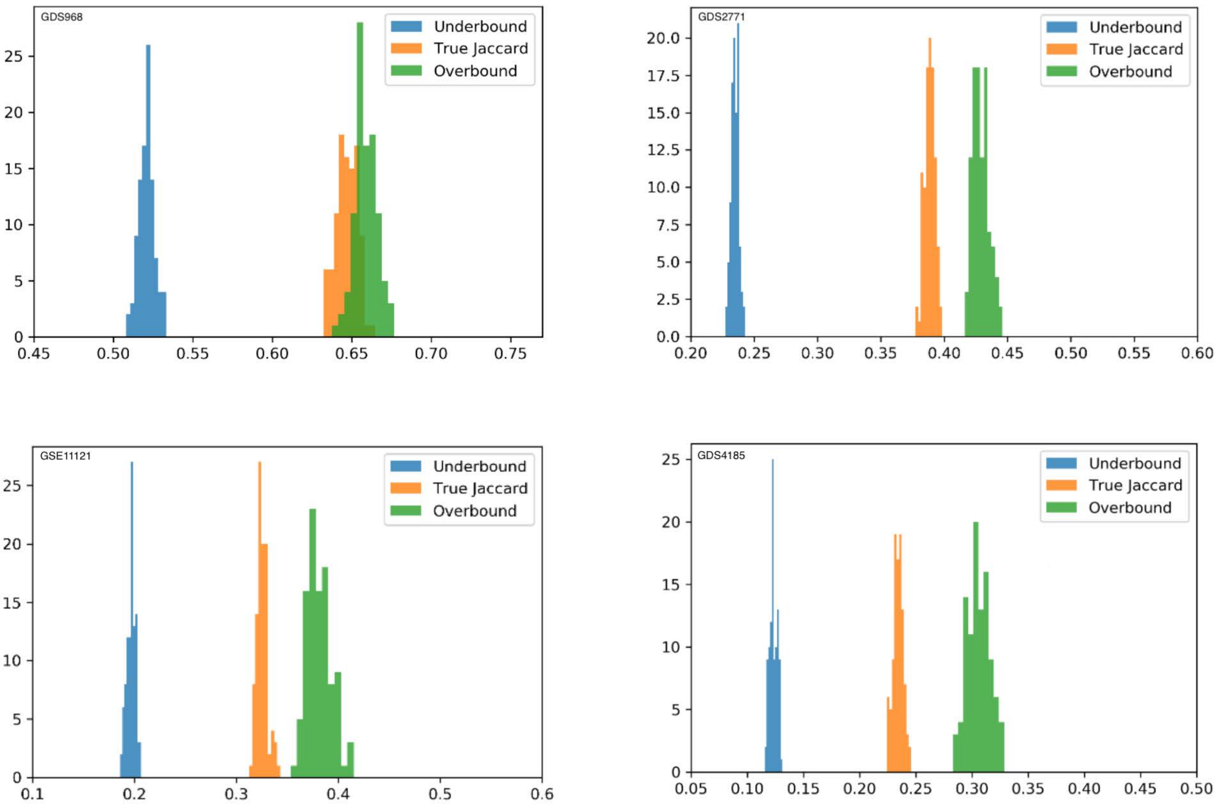

Fig D.5. Histograms for simulated datasets. Average underbound uRS, overbound oRS and true Jaccard histograms shown for 100 pairs of simulated datasets (for the true Jaccard) and 100 simulated datasets (for underbound and overbound), with distributions for each feature/outcome based on the empirical means and variances of features of 4 datasets { GDS968, GDS2771, GSE11121, GDS4185 }. Note that the x axis range is different for the different plots.

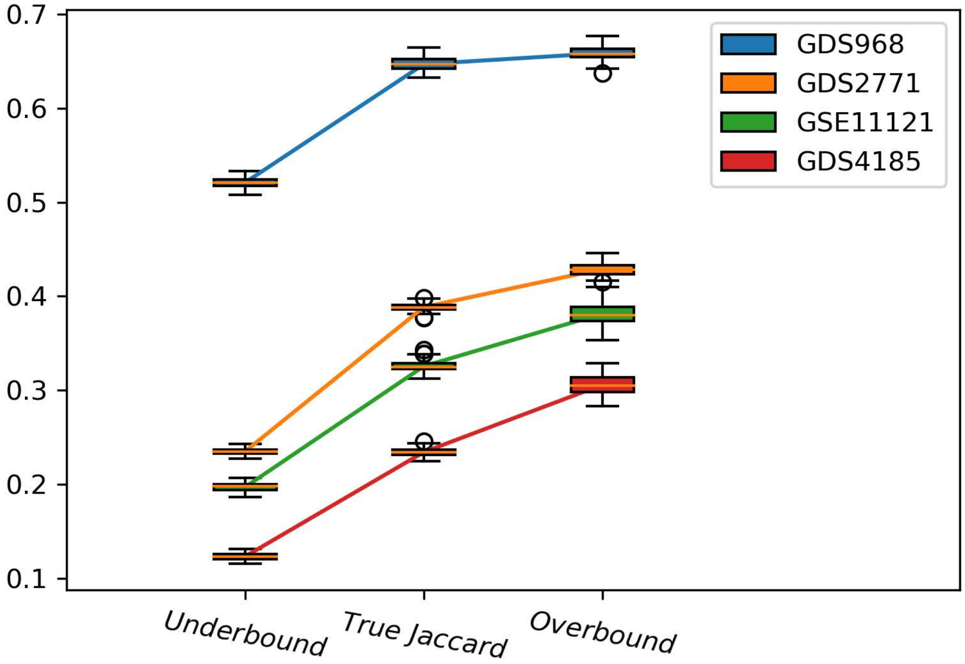

Fig D.6. Box-and-whiskers plots for simulated datasets. Average underbound uRS, true Jaccard $\widehat{\text{RS}}$, and overbound oRS values, for 100 pairs of simulated datasets (for the true Jaccard) and 100 simulated datasets (for underbound and overbound), with distributions based on the empirical means and variances of feature/outcome pairs of 4 datasets – using box-and-whiskers plots containing the median and IQR.

The second experiment explores Heuristic 7. For each distribution (corresponding to a dataset in $\rho \in \Gamma$), for each proportion $r \in \{ 0.1,\ 0.25,\ 0.5 \}$, we generate 100 pairs of datasets $\{[D1^i_{\rho,r},\ D2^i_{\rho,r}]\}_{i=1..100}$: For each $i \in \{1..100\}$, we first draw $n(\rho) \cdot r$ subjects – these will be in both datasets $D1^i_{\rho,r}$ and $D2^i_{\rho,r}$. We then complete each pair by drawing the remaining $n(\rho) \cdot (1 - r)$ subjects from the underlying distribution for $D1^i_{\rho,r}$, and then another $n(\rho) \cdot (1 - r)$ for $D2^i_{\rho,r}$. Hence, each of the ρ-datasets will have $n(\rho)$ subjects, and each $[D1^i_{\rho,r},\ D2^i_{\rho,r}]$ will share r proportion of the subjects in common.

Fig D.7 shows the Jaccard scores of each pair of datasets. We see that, as Heuristic 7 predicts, as the number of subjects in common between a pair of datasets increases, so does the average Jaccard score of the discovered-biomarker sets.

The third experiment explores Heuristic 9. In this experiment, we generate 100 pairs of datasets with $n = |D|$ subjects (same as the associated dataset D), 100 pairs with $n \times 2$ and 100 pairs with $n/2$ subjects, and compute the average true Jaccard score for the discovered biomarkers. Fig D.8 shows that, as Heuristic 9 predicts, as the number of subjects increases, the average Jaccard score increases as well.

Fig 4(a) earlier presented similar results, over 5 different datasets, but there taking (disjoint) subsets of the original dataset (rather than generating new subjects from the estimated distribution), and by presenting mean/variance values, rather than median/interquantile values, over 10 datbase-sizes (rather than 3). See also the black $\hat{RS}$ line in Fig 4(b), for yet another dataset.

D.4 Overlap between Two Multi-Sets

This subappendix explores some relevant theoretical properties of the oRS algorithm: Recall this algorithm partitions the "doubled version" DD of the original dataset D, into 2 size-n sub(multi)sets, $D1$ and $D2$. Below we show that, in expectation, one-half of the elements of D will appear in both both $D1$ and $D2$ – *i.e.*, $\mathbb{E}[\,|D1 \cap D2|\,] \ = \ n/2$. We then provide a natural notion of overlap between two multi-sets, and prove that the expected overlap between 2 random draws is 0, between the $D1$ and $D2$ defined above is 1/2, and between two bootstrap samples is 1.

Expected Overlap between Multi-Sets produced by oRS

Let $DD = \{a_1, b_1, a_2, b_2, \ldots, a_n, b_n\}$ denote a set of $2n$ distinct elements. Note $|D1 \cap D2|$ is the number of indices i such that exactly one of a_i and b_i is in $D1$. We provide a formal proof below. First, to provide the intuition: If the elements drawn in $D1$ were done independently, then clearly there a 50% chance that any element of DD will appear in $D1$ and a 50% chance that another element will *not* appear in $D1$. If these events were independent, then the chance that $a_i \in D1$ and $b_i \notin D1$ would be $P(\,a_i \in D1,\ b_i \notin D1\,) = \frac{1}{2} \times \frac{1}{2} = \frac{1}{4}$. Similarly, $P(\,a_i \notin D1,\ b_i \in D1\,) = \frac{1}{4}$. Hence, the chance that exactly one of $\{a_i, b_i\}$ is in A is
$P(\,a_i \in D1\ \oplus\ b_i \in D1\,) = P(\,a_i \in D1,\ b_i \notin D1\,) + P(\,a_i \notin D1,\ b_i \in D1\,) = \frac{1}{4} + \frac{1}{4} = \frac{1}{2}$.

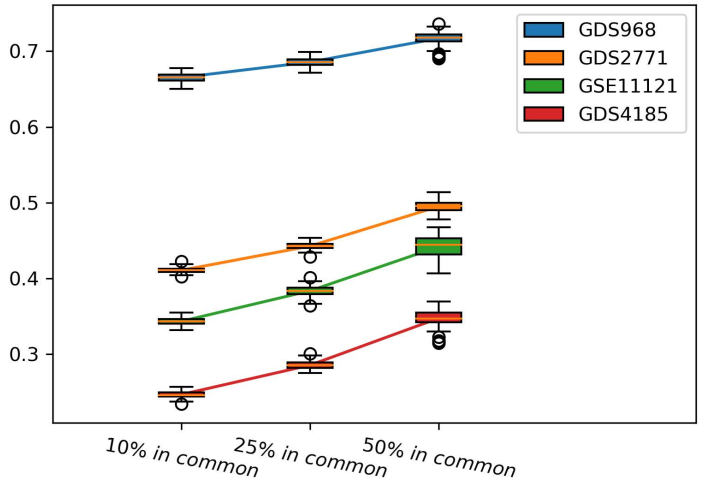

Fig D.7. Box and whiskers plot showing the average true Jaccard values for 100 pairs of simulated datasets for 4 different distributions, in 3 settings, where each pair has 10%, 25% or 50% of the subjects in common.

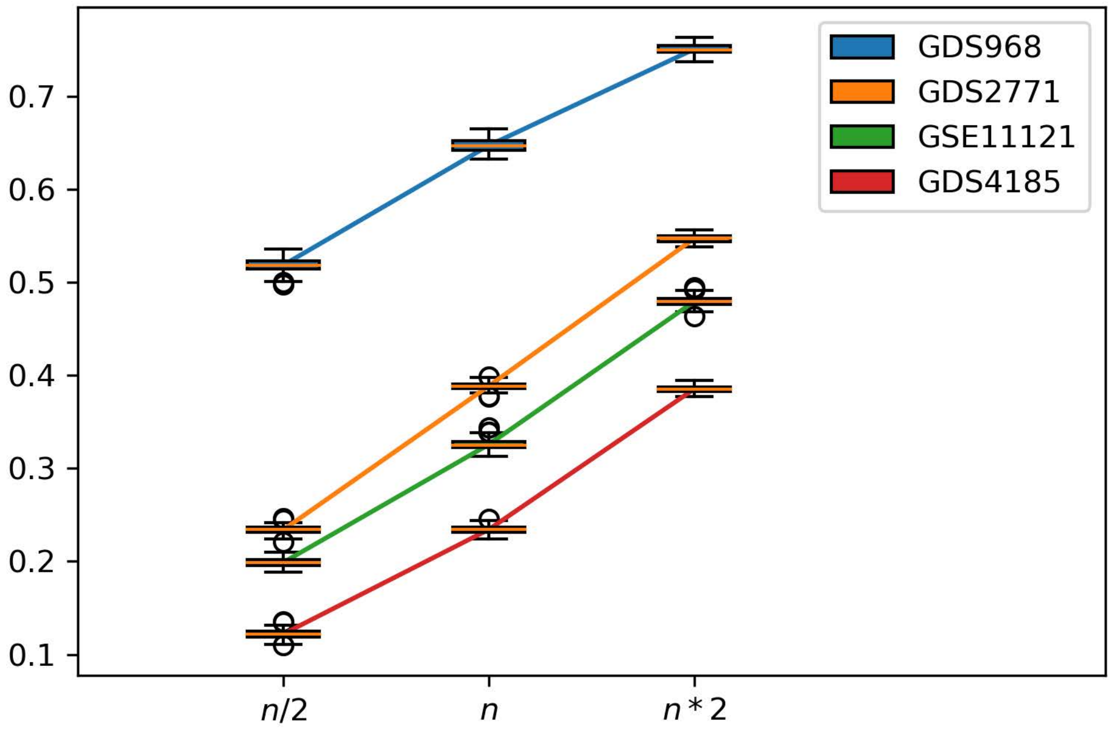

Fig D.8. Box and whiskers plot showing the average true Jaccard values for 100 pairs of simulated datasets for 4 different distributions, and for 3 different number of subjects (x-axis) relative to the original real datasets.

To be precise, we need to deal with the observation that the draws from DD are not independent. Now let A be a size-n subset drawn uniformly from DD (corresponding to the $D1$ mentioned above). Let $\mathcal{P}_n$ denote the set of all size-n subsets of DD, equipped with the uniform distribution. For any $A \in \mathcal{P}_n$, let $O_n(A)$ denote the number of indices $1 \le i \le n$ such that exactly one of a_i or b_i is in A, so $O_n : \mathcal{P}_n \to \mathbb{R}$ is a random variable. We view a fixed $A \in \mathcal{P}_n$ as the outcome of drawing a random size-n subset of DD. Note $|O_n|/n$ is the fraction of DD that is in both subsets.

Lemma 2 *Let O_n be defined as above. We have*

$$\lim_{n\to\infty} \frac{\mathbb{E}[\,O_n\,]}{n} \quad = \quad \frac{1}{2}$$

monotonically from above.

Proof Note that $\#\mathcal{P}_n = \binom{2n}{n}$, where $\#$ denotes the cardinality of a set.

Suppose we are given distinct elements $Z = \{z_1, \ldots, z_k\}$ where $k \le n$. We compute the probability that $\{z_1, \ldots, z_k\} \subset A$ where $A \in \mathcal{P}_n$ is a randomly chosen subset. Note that there is a bijection between size n subsets of DD containing $\{z_1, \ldots, z_k\}$ and size $n-k$ subsets of $DD \setminus Z$ where given a subset E of $DD \setminus Z$ of size $n-k$, we get the corresponding subset $E \cup Z$ of DD. Thus the number of elements in $\mathcal{P}_n$ containing Z is $\binom{2n-k}{n-k}$, where

$$\begin{aligned}
\frac{\binom{2n-k}{n-k}}{\binom{2n}{n}} \quad &= \quad \frac{\frac{(2n-k)!}{(n-k)!n!}}{\frac{(2n)!}{n!n!}} \quad = \quad \frac{\frac{(2n-k)!n(n-1)\cdots(n-k+1)}{n!n!}}{\frac{(2n)!}{n!n!}} \\
&= \quad \frac{(2n-k)!n(n-1)\cdots(n-k+1)}{(2n)!} \quad = \quad \frac{n(n-1)\cdots(n-k+1)}{2n(2n-1)\cdots(2n-k+1)}.
\end{aligned}$$

Moreover, the probability that Z is disjoint from a randomly chosen element of $\mathcal{P}_n$ is given by the same probability since for $A \in \mathcal{P}_n$, we have

$$Z \subseteq A \quad \Longleftrightarrow \quad Z \cap (DD \setminus A) \ = \ \{\}$$

and the map $A \mapsto (DD \setminus A)$ is a bijection from $\mathcal{P}_n$ to $\mathcal{P}_n$ since $\#A = n$.

In particular, for a fixed index $1 \le i \le n$, the probability that $\{a_i, b_i\}$ is disjoint from a random subset of DD of size n is

$$\frac{1}{4} \ \times \ \left(\frac{n-1}{n-\frac{1}{2}}\right). \tag{31}$$

Now, let S_j be the indicator random variable for the event that either a_j or b_j (or both) are selected, i.e. for a set $E \in \mathcal{P}_n$,

$$S_j(E) \quad = \quad \begin{cases} 1 \ : \ \ \{a_j, b_j\} \cap E \ \neq \ \{\} \\ 0 \ : \ \ \{a_j, b_j\} \cap E \ = \ \{\} \end{cases}$$

In particular, the number of distinct elements in E, denoted $D_n(E)$, is just given by $D_n(E) = \sum_{j=1}^{n} S_j(E)$. Thus by linearity of expectation and Eq 31, we have

$$\begin{aligned}
\mathbb{E}[\,D_n\,] &= \sum_{j=1}^{n} \mathbb{E}[\,S_j\,] = \sum_{j=1}^{n} \mathbb{P}(\{a_j, b_j\} \cap E \neq \emptyset) = \sum_{j=1}^{n} (1 - \mathbb{P}(\{a_j, b_j\} \cap E = \emptyset \\
&= \sum_{j=1}^{n} \left(1 \;-\; \frac{1}{4} \times \left(\frac{n-1}{n-\frac{1}{2}}\right)\right) \\
&= n\left(1 \;-\; \frac{1}{4} \times \left(\frac{n-1}{n-\frac{1}{2}}\right)\right)
\end{aligned}$$

Now to compute the expected overlap of the sets A and $DD \setminus A$ for $A \in \mathcal{P}_n$: If there are n_1 indices in A that occur exactly once, and n_2 indices that occur exactly twice, then

$$\begin{aligned}
n &= \#A = n_1 + 2n_2 \\
D_n(A) &= n_1 + n_2.
\end{aligned}$$

Since each index has two corresponding elements in DD, an index occurs in both A and $DD \setminus A$ if and only if it occurs exactly once in A. In particular, if $O_n(A)$ denotes the size of the overlap of A and $DD \setminus A$, we have $O_n(A) = 2D_n(A) - n$ and

$$\begin{aligned}
o_n := \mathbb{E}[\,O_n\,] &= 2\mathbb{E}[\,D_n\,] - n = 2n\left(1 - \frac{1}{4} \times \left(\frac{n-1}{n-\frac{1}{2}}\right)\right) - n \\
&= n\left(1 - \frac{1}{2} \times \left(\frac{n-1}{n-\frac{1}{2}}\right)\right) = n\left(\frac{n}{2n-1}\right).
\end{aligned}$$

Clearly $\lim_{n\to\infty} \frac{o_n}{n} = \frac{1}{2}$, as claimed.
■

Defining Overlap between Multi-Sets, in General Heuristic 7 stated that the Jaccard score of biomarkers found based on two datasets, depends on the overlap between those datasets. The standard definition of overlap applies to the sets that we considered so far. To deal with bootstrap sampling, we need to extend that definition. We also show that, with this extension, we see that our oRS model has a smaller overlap that bootstrap sampling, explaining our empirical results that oRS is better than bRS.

To illustrate, let $c(x, S)$ = the number of times that x appears in the (multi-)set S. For example, if

$$S_1 = [2\ 2\ 8\ 1\ 8\ 2\ 6\ 7\ 6\ 3]$$

then

$$c(1, S_1) = 1 \quad c(2, S_1) = 3 \quad c(3, S_1) = 1 \quad c(4, S_1) = 0$$

Assume $|S_1| = |S_2|$, and define:

$$\textsc{OverLap}(\,S_1,\,S_2\,) = \frac{1}{|S_1|}[\#(2 \in S_2) + \#(2 \in S_2) + \#(8 \in S_2) + \cdots + \#(3 \in S_2)] = \frac{1}{|S_1|}\sum_x c(x, S_1) \times c(x, S_2)$$

Lemma 3 *(a) If I_1 and I_1 are two size-n samples drawn independently from some continuous distribution over the reals (say from a mixture of Gaussians), then* $\mathbb{E}[\,\textsc{OverLap}(\,I_1,\,I_2\,)\,] = 0$

(b) If O_1 and O_1 are two size-n samples produced by the oRS algorithm, from a size-n sample D, then $\mathbb{E}[\,\textsc{OverLap}(\,O_1,\,O_2\,)\,] = \frac{1}{2}$.

(c) If B_1 and B_1 are two size-n boot-strap samples from a size-n sample D, then $\mathbb{E}[\,\textsc{OverLap}(\,B_1,\,B_2\,)\,] = 1$.

Proof: (a) follows by realizing that the probability of any real value appearing in two size-n draws is effectively 0.

(b) follows from the observation that we can partition $X = X_{12} \cup X_1 \cup X_2$, where X_{12} contains elements that appear exactly 1 time in each of O_1 and O_2, X_1 contains elements that appear 2 times in O_1 and 0 times in O_2, and X_2 contains elements that appear 2 times in O_2 and 0 times in O_1. Hence,

$$\begin{aligned}
\mathbb{E}[\,\textsc{OverLap}(\,S1,\,S2\,)\,] &= \frac{1}{n}\sum_{x\in X} c(x,O_1)\times c(x,O_2) \\
&= \frac{1}{n}\left[\sum_{x\in X_{12}} c(x,O_1)\times c(x,O_2) \quad + \quad \sum_{x\in X_1} c(x,O_1)\times c(x,O_2) \quad + \quad \sum_{x\in X_2} c(x,O_1)\times c(x,O_2)\right] \\
&= \frac{1}{n}\left[\sum_{x\in X_{12}} 1\times 1 \quad + \quad \sum_{x\in X_1} 2\times 0 \quad + \quad \sum_{x\in X_2} 0\times 2\right] \\
&= \frac{1}{n}\left[\mathbb{E}[\,|X_{12}|\,] \quad + \quad 0 \quad + \quad 0\right] \\
&= \frac{\mathbb{E}[\,|X_{12}|\,]}{|X|} \quad = \quad \frac{1}{2}
\end{aligned}$$

where the last line used Lemma 2, which proves that we expect X_{12} to contain half the elements of X.

(c) To prove (c),

$$\begin{aligned}
\mathbb{E}[\,\textsc{OverLap}(\,B_1,\,B_2\,)\,] &= \frac{1}{n}\sum_{x\in X} \mathbb{E}[\,c(x,B_1)\ \times\ c(x,B_2)\,] \\
&= \frac{1}{n}\sum_{x\in X} \mathbb{E}[\,c(x,B_1)\,]\ \times\ \mathbb{E}[\,c(x,B_2)\,] \qquad (32) \\
&= \frac{1}{n}\sum_{x\in X} 1\ \times\ 1 \qquad (33) \\
&= \frac{1}{n}\times|X| \quad = \quad 1
\end{aligned}$$

where Line 32 follows as B_1 and B_2 are independent bootstrap samples from X. To prove Line 33, note that $\sum_{x\in X} c(x,B) = n$ as some value appears in each position of B $\Rightarrow\ \ \mathbb{E}[\sum_{x\in X} c(x,B)\,] = n$.

Also $\mathbb{E}[\,c(x_a,B)\,]\ = \mathbb{E}[\,c(x_b,B)\,]\quad \forall x_a, x_b \in X$ by symmetry
$\Rightarrow\ \ n\ =\ \mathbb{E}[\,\sum_{x\in X} c(x,B)\,]\ \ =\ \ n\times\mathbb{E}[\,c(x,B)\,]\ \ \Rightarrow\ \ \mathbb{E}[\,c(x,B)\,]\ =\ \frac{n}{n}\ =\ 1$.
■

References

1. Strimbu K, Tavel JA. What are biomarkers? Current Opinion in HIV and AIDS. 2010;5(6):463.
2. Satagopan JM, Venkatraman E, Begg CB. Two-stage designs for gene–disease association studies with sample size constraints. Biometrics. 2004;60(3):589–597.
3. Benjamini Y, Hochberg Y. Controlling the false discovery rate: a practical and powerful approach to multiple testing. Journal of the royal statistical society Series B (Methodological). 1995; p. 289–300.

4. Benjamini Y, Yekutieli D. The control of the false discovery rate in multiple testing under dependency. Annals of statistics. 2001; p. 1165–1188.

5. Dunn OJ. Multiple comparisons among means. Journal of the American statistical association. 1961;56(293):52–64.

6. Hochberg Y. A sharper Bonferroni procedure for multiple tests of significance. Biometrika. 1988;75(4):800–802.

7. Holm S. A simple sequentially rejective multiple test procedure. Scandinavian journal of statistics. 1979; p. 65–70.

8. Zou J, Hao C, Hong G, Zheng J, He L, Guo Z. Revealing weak differential gene expressions and their reproducible functions associated with breast cancer metastasis. Computational biology and chemistry. 2012;39:1–5.
